# Hyper‐Thick Electrodes for Lithium‐Ion Batteries Enabled by Micro‐Electric‐Field Process

**DOI:** 10.1002/advs.202413444

**Published:** 2024-12-25

**Authors:** Tazdik Patwary Plateau, Gracie Boyer, Jonghyun Park

**Affiliations:** ^1^ Department of Mechanical and Aerospace Engineering Missouri University of Science and Technology Rolla MO 65409 USA; ^2^ Department of Electrical and Computer Engineering Missouri University of Science and Technology Rolla MO 65409 USA

**Keywords:** active materials particle arrangement, electric‐field casting, electrode structure engineering, hyper‐thick electrodes, micro‐macro diffusion path design

## Abstract

Increasing electrode thickness is a key strategy to boost energy density in lithium‐ion batteries (LIBs), which is essential for electric vehicles and energy storage applications. However, thick electrodes face significant challenges, including poor ion transport, long diffusion paths, and mechanical instability, all of which degrade battery performance. To overcome these barriers, a novel micro‐electric‐field (μ‐EF) process is introduced that enhances particle alignment during fabrication with reduced distance between anode and cathode. This process produces hyper‐thick (≈700 µm) electrodes with low tortuosity and improved ion diffusion. The μ‐EF electrodes achieve high areal capacities (≈8 mAh cm^−2^), while maintaining power density and long cycle life. The electrodes show stable performance under high C‐rate cycling and retain structural integrity after 1000 cycles at 2 C. By offering a scalable solution to the challenges of thick electrode fabrication, the μ‐EF process represents a significant advancement for high‐capacity LIBs in electric vehicles and energy storage systems.

## Graphical abstract

Thickening electrode is pivotal for increasing energy density in lithium‐ion batteries, yet challenges such as ion transport/diffusion limitations persist. Addressing these, an innovative macro‐/micro‐controlled micro‐electric‐field (μ‐EF) process enables hyper‐thick (≈700 µm) electrodes with reduced tortuosity, delivering exceptional areal capacities (≈8 mAh cm^−2^) and prolonged cycle life.

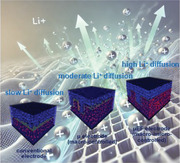

## 1 Introduction

The sustainability of our planet is closely tied to reducing carbon emissions through the electrification of transportation, particularly electric vehicles (EVs), as well as other sectors like energy storage and grid stabilization. Achieving this reduction requires the development of batteries with high energy and power densities for these diverse applications. Efforts to create various types of batteries, including lithium‐ion, sodium‐ion, zinc‐air, lead‐acid, nickel‐metal, and nuclear atomic batteries, have been successful.^[^ ^1^ ^]^ Among these, lithium‐ion batteries (LIBs) are particularly favored for their high energy and power density, as well as their safety and durability.^[^ ^2^ ^]^ While many advances in LIBs have focused on incorporating new materials, another critical factor in improving battery performance is the structure of the electrodes. One important strategy for increasing energy storage is to increase the thickness of the electrodes, allowing more active material to be loaded in a given area. However, increasing electrode thickness presents significant challenges such as longer ion diffusion paths, reduced electron transport, and heightened internal stress during cycling, all of which can compromise battery power performance and mechanical stability.^[^ ^3^, ^4^, ^5^, ^6^ ^]^

Recent efforts have focused on addressing the challenges of thickening electrodes, but the outcomes have been less successful than anticipated. For instance, a hybrid cathode with a 600 µm thickness, combining NMC‐811 (LiNi_0.8_Co_0.1_Mn_0.1_O_2_) with a solid‐state electrolyte, failed to achieve long cycle life due to only 30% of the structure being composed of active material.^[^ ^3^ ^]^ Another approach involved a 500 µm thick electrode, created via a freeze‐drying method using gum binder and single‐walled carbon nanotubes (SWCNTs), which showed a high mass loading of 511 mg cm^−2^ and an areal capacity of 79.3 mAh cm^−2^. However, this design suffered from severe degradation after just one cycle and ultimately failed after 15 cycles.^[^ ^7^ ^]^

One strategy to overcome these limitations is the use of 3D electrode geometries.^[^ ^3^ ^]^ These 3D structures provide short diffusion paths (SDPs) for ions, characterized by straight, non‐winding paths that promote faster ion transport and increase the interfacial area between anode and cathode, enhancing electrochemical reaction sites.^[^ ^5^ ^]^ By adopting 3D geometries with SDP characteristics, it becomes possible to develop thicker electrodes with increased active material content while maintaining excellent battery performance.^[^ ^5^ ^]^ Various techniques have been explored to create SDP electrodes, from the micro‐scale, which focuses on particle shapes and pore structures, to the macro‐scale, addressing local porosity uniformity.^[^ ^5^, ^7^, ^8^, ^9^, ^10^, ^11^, ^12^ ^]^

However, the key challenge in implementing these 3D‐structured electrodes lies in the fabrication methods. Traditional tape‐casting methods, widely used for fabricating LIB electrodes, face limitations when producing thick electrodes with a high material loading.^[^ ^5^, ^8^, ^9^ ^]^ Recent advancements in 3D printing technologies offer a promising alternative. Aerosol jet printing^[^ ^11^ ^]^ allows for the creation of intricate geometrical designs by depositing fine streams of material, offering precise control over material placement and enabling the fabrication of complex 3D‐structured electrodes. This method is well‐suited for flexible processing but requires careful control over parameters like jet pressure, atomization power, and aerosol flow to achieve optimal electrode structure.^[^ ^11^, ^13^, ^14^ ^]^ On the other hand, extrusion‐based 3D printing^[^ ^8^ ^]^ has demonstrated particular promise in significantly enhancing battery performance. This method extrudes material through a nozzle to create thick, hybrid 3D‐structured electrodes with SDPs. These structures provide a larger interfacial area for faster ion diffusion, leading to improved power output (2.3 mW cm^−2^) and energy density (64.6 J cm^−2^) compared to traditional laminated electrodes.^[^ ^5^, ^8^ ^]^ Despite these advancements, most additive manufacturing efforts have focused on micro‐batteries, with limited consideration for how processing affects the distribution of active particles and pore structure.^[^ ^10^, ^15^, ^16^, ^17^, ^18^ ^]^ Additionally, 3D printing methods remain time‐consuming and costly, presenting further challenges to scalability and practical implementation.^[^ ^14^, ^19^, ^20^, ^21^, ^22^ ^]^

Our previous study introduced a micro‐casting (µ‐casting) process to address these limitations by creating SDP 3D‐structured LIB electrodes.^[^ ^5^ ^]^ This technique uses a patterned 3D doctor blade during tape casting to form 3D‐patterned electrodes. The µ‐casting process offers several advantages: 1) it enhances active material utilization by reducing the distance between anode and cathode particles, 2) it enables the production of ultra‐thick electrodes (≈280 µm) with higher mass loading for greater specific and areal capacity, 3) it establishes structural integrity between 3D‐structured anodes and cathodes, allowing for interdigitated designs, and 4) it is a more cost‐effective and straightforward method compared to other 3D electrode fabrication techniques. The ultra‐thick electrode was defined as the electrodes having a thickness from 140 to 300 µm after drying whereas conventional thickness of the electrodes was ≈ 40–60 µm after drying. In that study,^[^ ^5^ ^]^ we successfully fabricated ultra‐thick NMC‐811 cathodes and MCMB anodes (with a mass loading of 35.73 mg cm^−2^) using the μ‐casting process, demonstrating substantial improvements over conventional laminated battery electrodes.

However, the µ‐casting process has certain limitations, including poor performance in electrodes thicker than 300 µm (ultra‐thick)^[^ ^5^ ^]^ and delamination from the current collector due to the thick electrodes. Specifically, the 300 µm limitation in the µ‐casting process underscores the constraints of macro‐level control. To overcome these limitations, we incorporate particle‐level control into the µ‐casting process. The conventional, uncontrolled particle distribution—where particles are randomly arranged—restricts ion and electron transport, results in a nonuniform concentration distribution, and can isolate particles, forming inactive zones. In this study, we developed a new method called micro‐electric‐field (µ‐EF) process, where an electric field (EF) is integrated into the µ‐casting process. This technique combines a patterned doctor blade with the application of high voltage during casting to control the arrangement of active material particles.

The µ‐EF process enhances ion transport by aligning particles to create low tortuosity and SDPs. Due to improved particle arrangement, the Li^+^ ions could find more straight‐forward travel path during ionic diffusion enabling low tortuosity.^[^ ^3^ ^]^ It also ensures even particle distribution and minimizes local porosity, effectively reducing the formation of inactive zones. These synergetic impacts enable the production of electrodes with a thickness of 700 µm defined as hyper‐thick electrodes (definition of Ultra‐thick and Hyper‐thick electrodes could be found in the scalebar showing in Figure S15, Supporting Information). This paper provides an in‐depth examination of the µ‐EF fabrication process, focusing on how the µ‐EF technique enables the creation of hyper‐thick electrodes (cathode – NMC 622, LiNi_0.6_Mn_0.2_Co_0.2_O_2_ and anode – MCMB, MesoCarbon Microbeads) and its relationship with key battery performance parameters such as morphology, porosity, conductivity, charge transfer properties, impedance, C‐rate capabilities, and cycling performance. A particular emphasis is placed on overcoming the challenges associated with drying these hyper‐thick electrodes while maintaining structural integrity. Additionally, we examine defect formation in cycled electrodes and assess their mechanical properties, enhancing our understanding of their performance and durability. The µ‐EF process represents a major breakthrough in battery technology, enabling the fabrication of significantly thicker, more efficient electrodes with improved energy density and charge transfer capabilities. This method holds the potential to transform energy storage across applications from portable electronics to electric vehicles and renewable energy systems.

## 2 Results and Discussion

**Figure 1:**
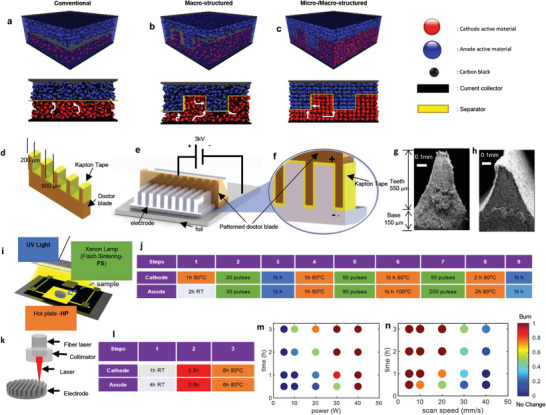
Schematic diagram of particle arrangement in the electrodes showing an assembly at different configurations; a) the conventional electrode design, b) macro‐structured electrode design, c) macro‐micro‐structured electrode design. The 3D view and cross‐sectional view depicts particles arrangement in these three configurations where facile Li^+^ ion movement (white arrows) is enabled in micro/macro‐structure. A modified patterned doctor blade with Kapton tape (d) was used to fabricate this electrode using novel micro‐EF process e,f) where high voltage of 3 kV was used. The SEM (scanning electron microscopy) images of the cross‐section of the μ‐EF electrode showing the height of the teeth and base (g) and a 3D view of the μ‐EF electrode (h). The drying process of the electrodes utilizing flash sintering (i) was optimized and achieved heating profile (j). Another route of drying using laser drying method (k) was also developed via a heating profile (l) and further optimized the laser power (m) and scan speed (n). The color‐bar shows the electrode status where blue means “no change” and red means “burn”.

The limitations of both conventional and our previous μ‐electrodes primarily stem from the random arrangement of particles within the electrode structure, caused by the uneven distribution of particles during the casting and drying processes. This non‐uniform distribution of active materials results in isolated particles, inconsistencies in the conductive network, irregular porosity, and the formation of inactive regions within the electrodes.^[^ ^9^ ^]^ In **Figure** **1**a–c, the schematic depicts the arrangement of active material particles (anode and cathode), carbon black, and binder in conventional, macro‐structured, and micro‐/macro‐structured electrodes. The macro‐structured electrode refers to our previously developed patterned electrode, while the micro‐/macro‐structured electrode represents a particle arrangement‐controlled design with simultaneous macro‐structuring, which is the focus of this study. As the thickness of electrodes increases, the conventional design results in higher tortuosity, leading to longer diffusion paths for Li^+^ ions.^[^ ^3^, ^5^, ^23^ ^]^ While macro‐structured electrodes provide SDPs for Li^+^ ions, the random arrangement of particles still limits their overall efficiency. Our new µ‐EF process‐based micro‐/macro‐structured electrodes address these limitations by improving particle arrangement, eliminating isolated particles, inactive regions, and random conductive networks, while enhancing ionic conductivity.

Although the concept of micro‐/macro‐structured electrodes sounds promising, the practical challenge lies in fabricating these structures. We overcame this challenge by applying a high EF during the µ‐casting process, using the same patterned doctor blade developed in our previous work,^[^ ^5^ ^]^ as shown in Figure 1d. To prevent short‐circuiting during the EF application, the blade was covered with Kapton tape. The setup is illustrated schematically in Figure 1e,f, where a high voltage of 3 kV was applied using a power supply. The EF applied during casting caused the active material particles to align, resulting in improved particle arrangement.^[^ ^9^ ^]^ Moreover, in the slurry system, the electric field can influence these particles through alternative mechanisms, such as charge redistribution or particle‐medium electrostatic interactions, facilitating their alignment. These combined effects, including dielectric polarization and electrostatic interactions, likely explain the observed structural changes.^[^ ^8^, ^23^, ^24^ ^]^ Therefore, the particle alignment is driven by a combination of dielectric polarization, charge interactions, and electrostatic forces, rather than purely classical dielectric behavior. To verify the effect of the EF on particle distribution, a test using a diluted slurry was performed. The results showed that the particles aligned along the direction of the applied EF due to induced dipoles. Further details are presented in Note S1 (Supporting Information), which confirms that a 3 kV electric field successfully arranged the electrode material particles, as illustrated in Figure S1 (Supporting Information).

Next, the actual slurry was used to fabricate the electrodes. The cross‐sectional view of the µ‐EF electrode, shown in Figure 1g, indicates the height of the teeth (550 µm) and the base (150 µm). A 3D view of the electrode is provided in Figure 1h. One key challenge in fabricating hyper‐thick electrodes (>500 µm) is maintaining mechanical stability during the fabrication and drying processes. Issues such as delamination from the current collector, structural collapse, and electrode breakage or cracking present significant obstacles. To address delamination, two approaches were implemented: binder pre‐drying at 95 °C for 10 h and acid etching of the current collector with 5% HCl (details provided in Note S2, Supporting Information). Pre‐drying the PVDF (polyvinylidene fluoride) binder improved adhesion properties,^[^ ^24^ ^]^ while acid etching increased the surface roughness of the current collector. This synergistic combination effectively resolved the delamination issue.

However, as electrode thickness increases further, the drying process becomes more challenging. The conventional oven heating method (overnight at 120 °C in a vacuum) created a thermal gradient across the electrode's cross‐section, leading to deformation, cracks, and fractures. To resolve these issues, two alternative drying methods were developed and tested: the Flash Sintering‐influenced drying method (Figure 1i,j) and the Laser‐influenced drying method (Figure 1k,l). Both methods offered rapid drying. The heating profiles for both methods were optimized by evaluating key process parameters. The final profiles are shown in Figure 1j for the flash sintering method and Figure 1l for the laser drying method. However, continuous flash sintering resulted in burning and crack formation in the electrodes (Figure S2, Supporting Information), while the laser drying method caused burning and the formation of carbonized char. Figure 1m,n illustrates the impact of laser power and scan speed on electrode burning, with green‐colored values indicating optimal results. A combination of 30 W laser power and a scan speed of 30 mm s^−1^ was found to be the most efficient, completing the drying process in 30 min, and was selected for further experiments. Here, we found that, although the same solvent evaporation method was applied, distinct approaches were required for each electrode type.

### 2.1 Effect of Electric Field on μ and μ‐EF Process

**Figure 2:**
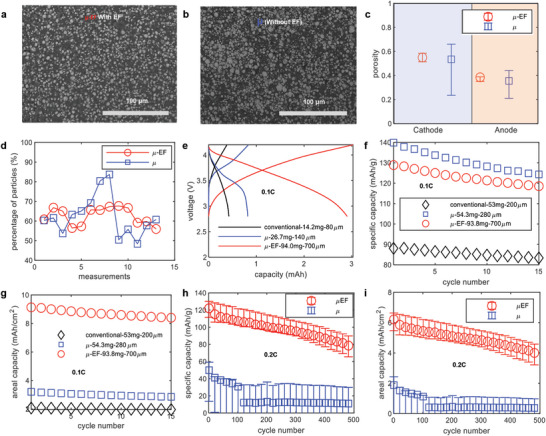
SEM image of the μ‐EF electrode showing the particle arrangement (a), SEM image of the μ electrode showing the particle arrangement (b), porosity analysis of the μ and μ‐EF anode and cathode evaluated by using ImageJ software (c), and measurement of the percentage of the particles in different measurements on the SEM images showing wide variation in the μ electrodes than the μ‐EF electrodes (d). Voltage profile comparing conventional, μ and μ‐EF full cells using NMC‐622 cathode and MCMB anode (e), the specific (f) and areal capacity (g) differences of μ and μ‐EF full cells at 0.1 C (h). More cells for the μ and μ‐EF full cells were used to analyze the impact of the EF on the electrodes via evaluation of the specific capacity (h) and areal capacity (i) at 0.2C where the electrodes had a thickness of 600 µm and their mass was ≈70–90 mg. From (c–i), the red circle marker indicates the data for μ‐EF electrodes, blue square marker indicates the data for μ electrodes, and black square marker indicates the data for conventional electrodes.

The impact of the EF on electrode morphology and electrochemical performance was investigated. Several scanning electron microscopy (SEM) images from different zones and samples were analyzed to assess particle distribution within the electrodes. **Figure** **2**a presents an SEM image of the μ‐EF electrodes (additional images are available in Figure S3, Supporting Information), while Figure 2b shows an electrode fabricated without the use of an EF (with more images in Figure S4, Supporting Information). Upon examining these figures, the particle distribution was not perfectly aligned due to the limited mobility of particles in the high mass loading paste, which is required to deliver sufficient energy, but this restricted particle movement under the EF.^[^ ^5^ ^]^ The particle distribution in electrodes with and without the EF was further evaluated by measuring porosity using ImageJ software.^[^ ^26^ ^]^ Both types of electrodes (with and without the EF) exhibited similar average porosity in the cathode and anode (Figure 2c). However, the μ electrodes showed a broader range of porosity values compared to the μ‐EF electrodes, and this trend was consistent for both the anode and cathode. Additionally, particle percentages (Figure 2d) were assessed from SEM images, revealing significant variability across different images. These findings suggest that particle arrangement in the μ electrodes was more random than in the μ‐EF electrodes.

A comparison of capacity for conventional, μ, and μ‐EF electrodes was conducted (Figure 2e). The μ‐EF electrodes showed a significantly higher capacity at 0.1C compared to both conventional and μ electrodes. This capacity increase can be attributed to the greater thickness (700 µm vs 140 µm) and mass of the μ‐EF electrode. To further validate these results, another set of cells was cycled for a longer duration (15 cycles), and both specific capacity and areal capacity were compared. The μ‐EF battery demonstrated 4.5 times higher areal capacity than the conventional battery. However, the specific capacity of the μ‐EF electrode did not exceed that of the μ electrode, likely due to its hyper‐thickness (Figure 2f,g). To investigate further, multiple cells with μ and μ‐EF electrodes (both with a thickness of 600 µm and a mass between 70–90 mg) were cycled at 0.2C for 500 cycles (Figure 2h,i) using the CR2032 coin cell format. The μ cells exhibited lower performance in both specific and areal capacity compared to the μ‐EF cells, indicating that the improved particle arrangement in the μ‐EF electrodes enhanced performance by enabling higher ionic diffusion with reduced tortuosity.

### 2.2 Effect of Drying Method on Morphology and Performance

**Figure 3:**
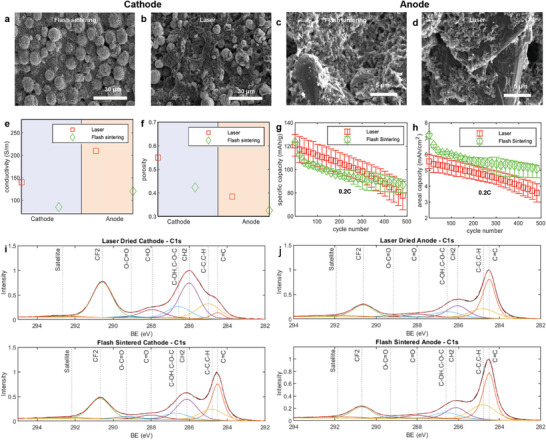
SEM images of the μ‐EF cathodes fabricated by flash sintering method (a) and laser drying method (b). The anode electrodes fabricated by flash sintering method (c), indicated by green diamond shape marker, and laser drying method (d), indicated by red square marker. A comparison of the electrical conductivity (e) and porosity (f) between the laser drying method and flash sintering method for anode and cathode was showed. The specific capacity (g) and areal capacity (h) of the μ‐EF cells formed via flash sintering drying method and laser drying method was compared (green circle marker indicated for flash sintering based drying data and red square marker was for laser drying data). XPS analysis of the electrode surfaces showing comparison of peak fitting of C1s signal for Laser‐dried (up) and Flash sintered (bottom) cathodes (i) and anodes (j) where BE means Binding Energy. Here different bonds were demonstrated in the plots and dotted lines indicated the BE values to the corresponding bonds.

As previously noted, a key challenge in the μ‐EF process is managing the drying stage to prevent thermal‐gradient‐induced cracks and delamination. To address this, two drying techniques—flash sintering and laser drying were utilized, both involving multiple steps (Figure 1i–l). It was crucial to determine which method was more effective for producing hyper‐thick electrodes. SEM images were used to conduct a morphological analysis of cathodes and anodes created via the laser and flash sintering drying processes. The cathode produced by flash sintering (**Figure** **3**a) showed uniform particles with binders present, while the cathode created using laser drying (Figure 3b) exhibited a porous structure and a burned PVDF binder surface. In the case of the anode, the laser‐dried electrode revealed more exposed graphite layers compared to the flash‐sintered one (Figure 3c,d). Additional SEM images comparing these two methods can be seen in Figures S5 and S6 (Supporting Information). The burning of the binder during laser drying may have contributed to an increase in the carbon content of the electrode. To evaluate the impact of these drying methods, the conductivity and porosity of the electrodes were analyzed. Laser‐dried electrodes exhibited higher conductivity and porosity compared to those dried by flash sintering (Figure 3e,f), and this trend was consistent for both anodes and cathodes. Porosity varied more significantly with laser drying than with flash sintering (Table S3, Supporting Information). The increased conductivity could be due to partial binder carbonization, while higher porosity may result from binder evaporation during carbonization. A further comparison of the two drying methods was made by examining the specific and areal capacities of the μ‐EF cells. In this test, all electrodes had the same thickness of 600 µm and a mass range between 70–80 mg. Results, based on tests of five cells, are presented in Figure 3g,h, showing specific and areal capacity with upper and lower limits. The flash sintering‐based μ‐EF cells demonstrated slightly lower specific capacity but experienced less capacity fading than the laser‐dried cells. The higher specific capacity in the laser‐dried electrodes could be due to mass reduction caused by binder evaporation. However, flash‐sintered μ‐EF cells exhibited higher areal capacity, suggesting that unwanted materials formed on the laser‐dried electrodes may have contributed to electrochemical reactions, leading to lower areal capacity and increased capacity fading. Another noteworthy observation was the high variability in the results from the laser‐dried electrodes, likely caused by differences in binder evaporation and carbonization. While laser drying proved effective for electrode fabrication, the substantial variations and unpredictable changes in electrode characteristics made it less suitable for producing hyper‐thick μ‐EF electrodes. Furthermore, the laser‐dried and flash‐sintering‐based electrodes were compared using X‐ray Photoelectron Spectroscopy (XPS) (Laser and Flash sintered cathode and anode C1s signal in Figure 3i,j, details are available at Note S3; Figures S12 and S13; Tables S4 and S5, Supporting Information) to attempt to observe any chemical composition changes in the electrode surface for the different drying processes. A larger C═C peak was observed in the Flash sintered cathodes.^[^ ^28^, ^29^, ^30^, ^31^, ^32^ ^]^ This may be attributed to the fusion of the carbon black particles. However, the dominating peaks for the Laser‐dried cathodes were the CH2 and CF2 peaks from PVDF while the C═C peak was comparatively smaller. This could indicate a combustion reaction of carbon black particles for the Laser‐dried cathode, resulting in CO2 evaporation and a lower C═C signal.

### 2.3 Electrochemical Performance of μ‐EF

**Figure 4:**
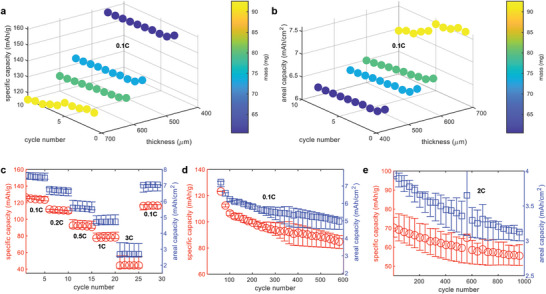
The electrochemical performance of the μ‐EF cells showing specific capacity results at 0.1C for 10 cycles of different thickness of the electrodes (a) and the areal capacity was showed in (b). Different colors in the marker of the plots represents the mass of the electrodes according to the color‐bar where blue indicates a value of 60 mg and yellow indicates a value of 95 mg. C‐rate performance results of the 700 µm μ‐EF electrodes showed at different C‐rates of 0.1, 0.2, 0.5, 1, 3, and 0.1 C (c), and long cycling of 600 cycles at low C‐rate of 0.1 C (d) and 1000 cycles at high C‐rate of 2 C showed (e). Blue square marker was used for the areal capacity showing on the right Y axis of the plots and red circle marker was used for the specific capacity showed in the left Y axis in the plots.

In this section, the impact of electrode thickness on electrochemical performance is analyzed. The primary goal of this work is to increase electrode thickness without significantly compromising power performance. To achieve this, four different μ‐EF electrode thicknesses (450, 550, 600, and 700 µm) were selected to study capacity. The results presented in **Figure** **4**a,b reflect the average performance of four cells at a low current (0.1C). As expected, increasing the thickness and mass of the electrodes led to a reduction in specific capacity but an increase in areal capacity. Notably, even with an exceptionally thick electrode of 700 µm, significantly thicker than the conventional 60–80 µm range,^[^ ^3^, ^5^ ^]^ the cells performed well, with no significant capacity degradation, which is a key issue typically associated with conventional thick electrodes.^[^ ^3^, ^23^, ^32^, ^33^, ^34^ ^]^ Furthermore, an impressive areal capacity of ≈8 mAh cm^−2^ was achieved at 0.1C with these hyper‐thick μ‐EF electrodes. Previous studies have highlighted difficulties in cycling at high C‐rates. To evaluate the performance of the 700 µm μ‐EF electrodes across a range of C‐rates, from low to high (0.1, 0.2, 0.5, 1, and 3 C, and back to 0.1 C), current was applied (Figure 4c). The results showed a clear reduction in both specific and areal capacity as the C‐rate increased, with more significant variations in capacity at higher C‐rates. This may indicate instability in the μ‐EF cells under high‐current charging and discharging conditions. However, unlike other thick electrode‐based cells that cannot cycle at a high C‐rate of 3C,^[^ ^8^, ^32^, ^33^, ^34^ ^]^ the μ‐EF cells demonstrated decent performance, achieving over 40 mAh g^−1^ specific capacity and ≈3 mAh cm^−2^ areal capacity. When compared to previous results for μ‐electrode‐based cells,^[^ ^5^ ^]^ the μ‐EF electrodes showed an ≈30% increase in specific capacity and an 86% improvement in areal capacity.

A major challenge with thick electrodes is their significant degradation during long‐term cycling due to severe capacity fading, which ultimately leads to cell failure.^[^ ^35^, ^36^, ^37^, ^38^ ^]^ To address this, long‐term cycling tests were conducted on the hyper‐thick μ‐EF electrodes, with the results shown in Figure 4d. All μ‐EF cells were cycled at 0.1C for 600 cycles, resulting in a 33% fade in both specific and areal capacities. However, none of the cells failed during this extended cycling. Additionally, the stability of the μ‐EF cells was tested at a high C‐rate of 2C over 1000 cycles (Figure 4e). The results showed a 22% reduction in specific capacity and a 26% reduction in areal capacity, with no cells failing before completing 1000 cycles. The organized particle arrangement in the low‐tortuosity μ‐EF electrodes allowed for efficient ionic movement, helping to minimize internal stress during cycling. This reduction in internal stress contributed to lower capacity degradation at high C‐rates and supported a longer battery lifetime.

### 2.4 Impedance Analysis and Diffusion

**Figure 5:**
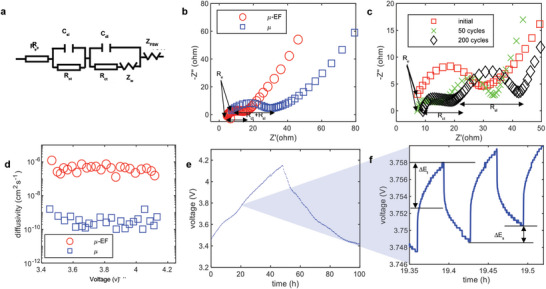
The circuit diagram used for the EIS analysis (a). A comparison showing in the Nyquist plot between the μ (blue square marker) and μ‐EF (red circle marker) cells (b). An evaluation of the Nyquist plot analyzing the impedance behavior of the μ‐EF cell upon long cycling where red square marker showing initial result, green cross marker showing result after 50^th^ cycle, and black diamond marker showing result after 200^th^ cycle (c). GITT test for observing the diffusivity of the μ (blue square marker) and μ‐EF (red circle marker) cells where diffusivity is in the logarithmic scale. The voltage profile of the electrodes where current was applied for 10 min and rested for 10 min for both charging and discharging (e) and zoom‐in voltage profile to show how the ∆E_s_ and ∆E_t_ were measured to calculated diffusivity (f).

The low tortuosity resulting from the particle arrangement is anticipated to enhance the diffusion properties of Li^+^ ions. To assess charge transfer resistance and ionic diffusion in the μ‐EF cells, electrochemical impedance spectroscopy (EIS) was conducted, comparing the performance of μ and μ‐EF cells. In a typical Nyquist plot,^[^ ^5^, ^39^, ^40^, ^41^, ^42^ ^]^ as depicted in **Figure** **5**a, the high‐frequency intercept on the Z' axis represents the electrolyte resistance (Re). The semicircle in the mid‐frequency range corresponds to the charge transfer resistance (R_ct_). An additional surface layer resistance (R_sl_) and surface layer capacitance (C_sl_) were considered to account for the surface interface.^[^ ^43^ ^]^ The Warburg impedance (Z_w_), shown as a sloping line at low frequencies, represents lithium‐ion diffusion over a semi‐infinite length. A finite space Warburg‐type element (Z_FSW_) was used to describe diffusion in a medium where the interface impedes species flow.^[^ ^5^, ^40^, ^44^, ^45^, ^46^ ^]^ Further details on the impedance analysis can be found in Note S4 (Supporting Information). The comparison between μ and μ‐EF cells (Figure 5b) revealed that the bulk resistance was higher in the μ cell compared to the μ‐EF cell. Instead of separate semicircles for R_ct_ and R_sl_, both cells exhibited a single combined semicircle, likely because the Solid Electrolyte Interphase (SEI) layer had not fully formed in the initial cycles.^[^ ^47^ ^]^ In μ‐EF cells, a reduction in charge transfer resistance was observed, indicating enhanced charge transfer. The Warburg impedance (Zw) in the μ‐EF cell was also 33.2% lower than in the μ cell, and the Z_FSW_ in the μ‐EF cell was only one‐fourth that of the μ cell (Table S6, Supporting Information). The slightly higher double‐layer impedance in the μ‐EF cell could be due to its larger surface area, which stores more charged particles, contributing to the double‐layer mechanism.^[^ ^48^ ^]^ Analysis of the Nyquist plots over increasing cycles (Figure 5c) showed that R_ct_ and R_sl_ became more distinguishable after 50 and 200 cycles. Both Rct and Rsl increased with cycling, indicating cell degradation due to reduced ionic transfer. The Warburg impedance also increased as the cycles progressed, as detailed in Table S7 (Supporting Information). C_dl_ and C_sl_ also increased, possibly due to the thickening of the SEI layer.^[^ ^47^, ^48^ ^]^ To further explore diffusion behavior, a galvanostatic intermittent titration technique (GITT) test was performed (Figure 5d), similar to prior studies.^[^ ^5^, ^49^, ^50^ ^]^ A very low C‐rate of 0.01 C was used, along with a 10‐min resting period between steps; additional details can be found in Note S5 (Supporting Information). The voltage profile of the μ‐EF cell is shown in Figure 5e, with a zoomed‐in image displaying the voltage differences for ∆E_t_ and ∆E_s_ in Figure 5f. The diffusion coefficient was calculated using Equation S1 (Supporting Information), revealing that the diffusivity of the μ‐EF electrodes was ≈2 orders of magnitude higher than that of the μ electrodes. This conclusion aligns with the results from the impedance analysis.

### 2.5 Defect Analysis & Mechanical Properties

**Figure 6:**
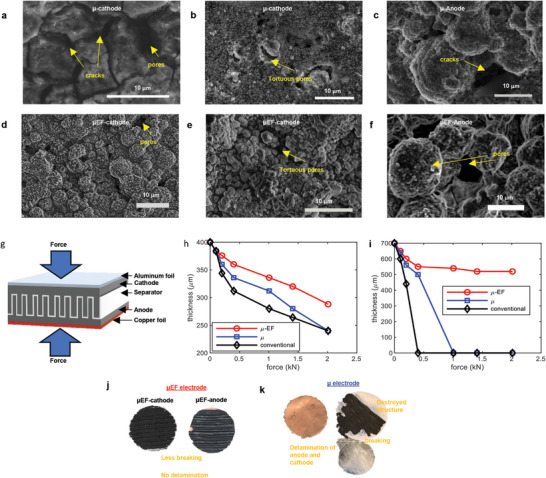
SEM images of cycled μ electrodes after 500 cycles at 0.2 C – cathode (a,b) and anode (c). SEM images of cycled μ‐EF electrodes after 500 cycles at 0.2 C – cathode (d,e) and anode (f). Yellow writing on the figures indicated different defects of the electrodes. The assembly of the anode and cathode including separator and current collectors (aluminum foil for cathode and copper foil for anode) showing the testing process of the mechanical properties of the electrodes (g). The mechanical compression analysis result for the electrodes (red circle marker for μ‐EF, blue square marker for μ and black diamond marker for conventional electrodes) were illustrated for 400 µm thick samples (h) and 700 µm thick samples (i). The digital photographic images of the electrodes after mechanical compression analysis – μ‐EF anode and cathode (j) and μ anode and cathode (k). Yellow‐colored writing indicated the observed status of the electrodes.

One key degradation mechanism in electrodes is the repeated stress caused by volume changes during intercalation and deintercalation. This generated stress becomes more severe where a concentration gradient develops inside the particles during continuous cycling, resulting in defects such as cracks, pores, and potential breakage. To assess these defects, cells cycled for 500 cycles were opened and analyzed using SEM for both μ and μ‐EF electrodes. **Figure** **6**a,b shows images of μ‐cathodes, and Figure 6c shows a μ‐anode, while Figure 6d,e depict μ‐EF cathodes, and Figure 6f displays a μ‐EF anode. The results clearly indicate that μ‐cathodes experienced more degradation than μ‐EF cathodes. The μ‐cathodes exhibited large cracks, some extending up to 10 µm, and pores ranging from 1–8 µm in diameter. In contrast, the μ‐EF cathodes showed no cracks and only small, tortuous pores less than 2 µm in size. These findings align with diffusion analysis, which showed that μ‐cells had lower diffusivity compared to μ‐EF cells. The improved particle arrangement and distribution in the μ‐EF electrodes allowed for shorter diffusion paths and facilitated easier ionic movement during charging and discharging. This low‐tortuosity design enhances electrolyte permeability and ion transfer rates over 500 cycles, reducing the concentration gradient and associated internal stress, leading to less physical degradation of the electrodes.

Additionally, the structural integrity of hyper‐thick μ‐EF electrodes was examined, as this is critical for designing reliable thick electrodes. Mechanical testing was performed to assess the robustness of μ‐EF electrodes compared to conventional μ electrodes. The test involved assembling the anode, cathode, separator, and current collector, and applying pressure from both the top and bottom to simulate assembly conditions (Figure 6g). For 400 µm thick electrodes (Figure 6h), μ‐EF electrodes demonstrated superior mechanical properties compared to μ and conventional laminated electrodes, showing less thickness reduction under loads from 0 to 2 kN, indicating greater resistance to mechanical compression and pressure. When the electrode thickness was increased to 700 µm (Figure 6i), the mechanical advantages of μ‐EF electrodes became even more pronounced. Under a 2 kN load, μ‐EF electrodes maintained stable mechanical performance, while μ electrodes failed, exhibiting severe delamination and damage (Figure 6k). Visual inspection revealed that, despite minor breakage, μ‐EF electrodes (Figure 6j) retained their overall structure, demonstrating enhanced mechanical strength due to the well‐ordered particle arrangement. This suggests that μ‐EF electrodes have superior mechanical integrity, making them ideal for practical battery manufacturing and real‐world applications. The organized particle distribution helps to minimize internal damage caused by mechanical stress, making this approach promising for high‐capacity lithium‐ion batteries, which require thick electrodes to meet energy and power demands while ensuring long‐term reliability and stability.

## 3 Discussion

The μ‐EF electrodes represent a breakthrough in battery technology by achieving hyper‐thick (700 µm) electrodes without sacrificing power performance. They offer superior diffusivity and reduced stress generation, which, combined with enhanced charge transfer enabled by the micro‐macro architecture, resulted in exceptional cycle life and stable capacity. An areal capacity of ≈8 mAh cm^−2^ was achieved by arranging the active material particles in a well‐ordered pattern, creating low‐tortuosity paths for better ion movement, while the patterned doctor blade facilitated shorter diffusion paths. Furthermore, a comparison of the areal capacity of the thick electrodes in recent literatures utilizing SDP structures has been observed in Figure S16a (Supporting Information). Despite their increased weight and thickness, the µEF electrodes demonstrated superior performance compared to the other configurations. Another critical parameter for lithium‐ion batteries (LIBs) is the volumetric energy density. Although the electrode‐level volumetric energy density of the µEF electrodes was lower than that of conventional thin electrodes (60–80 µm),^[^ ^8^ ^]^ as depicted in Figure S16b (Supporting Information), the cell‐level volumetric energy density was higher, showed in Figure S16c (Supporting Information). This improvement is attributed to the enhanced utilization of active materials and a reduction in the volume of non‐active components, such as separators and the aluminum and copper current collectors. This organized particle distribution not only improved electrochemical properties but also enhanced mechanical strength, ensuring structural integrity during assembly. This innovative study highlights the potential of thick electrodes for electric vehicle batteries, showcasing impressive capacity and mechanical durability. The work successfully demonstrates theoretical hypotheses through comprehensive experimental analysis across morphological, electrical, mechanical, and electrochemical dimensions.

## 4 Experimental Section

### Materials and Paste Preparation

This study followed the same recipe for making the paste of electrodes consisting active materials, binder, conductive component, and solvent as described in our previous report.^[^ ^5^ ^]^ The cathode was fabricated using a LiNi_0.6_Co_0.2_Mn_0.2_O_2_ (NMC‐622) paste, while the anode was made of the active material – Meso Carbon Micro Beads (MCMB). The cathode pastes were prepared by mixing 85.5 wt.% NMC‐622 powder (purchased from Linyi Gelon LIB Co.Ltd., particle size: 13 µm) with 6.5 wt.% carbon black (CB, obtained from Alfa Aesar) as the conductive agent and 8 wt.% Polyvinylidene fluoride (PVDF, sourced from Sigma‐Aldrich) as the binder. This mixture was dissolved in N‐Methyl‐2‐pyrrolidone solvent (NMP, purchased from Sigma‐Aldrich). For the anode pastes, 85 wt.% MCMB powder (obtained from MTI Corp., particle size: 17.65 µm) was combined with 5 wt.% Carbon Black (CB, from Alfa Aesar) and 10 wt.% PVDF. The powders were dissolved in NMP solvent. To homogenize the pastes, a Speed Mixer (Flack Teck Inc.) was used to mix them at 3500 RPM for 20 min at room temperature.

### Preparation of Current Collector and Binder

A pre‐drying procedure was implemented before the utilization of the binder PVDF powder in the slurry preparation. This pre‐drying step involved subjecting the powders to a 10‐h drying process at 95 °C in a vacuum oven. For the anode and cathode, the current collectors were utilized as the substrate of the electrodes. The anode's current collector was a 10 µm thick copper foil, while the cathode's current collector was a 12.5 µm thick aluminum foil. To enhance the adhesion of the electrode materials to the current collector, an acid etching process was employed. Various percentages of different acids were tested, and the most effective result was achieved using a 5% HCl (hydrochloric acid) solution for a duration of 5 min. This acid etching method was consistently applied to all the electrodes fabricated in this study. Further information on the preparation and optimization can be found in Note S2 (Supporting Information).

### μ‐EF Casting Process

A patterned doctor blade (Figure 1d) was made by cutting 304 stainless steel plate using EDM (energy Dispersive Machining, SODICK, model‐ LN2 W) method similar to our previous work.^[^ ^5^ ^]^ The pattern was made considering a clearance thickness of the Kapton tape. The Kapton tape thickness was 50 µm and so a gap between the threads were 700 µm which can result in a final gap of 600 µm for the teeth and 200 µm for the base width as shown in Figure 1d. A high electric field was employed in this μ‐EF casting process which was obtained by a high‐voltage power supply (XP power). In particular, the positive terminal was connected with the doctor blade and the negative terminal was connected with the substrate foil (showed in Figure 1e,f). A high voltage of 3 kV was controlled in the system by using a computer software LABVIEW and was measured by using a high‐impedance‐high‐voltage meter (ES105, ESDEMC) for confirmation. A voltage higher than 3 kV resulted in spark formation and so higher voltage was not used in the μ‐EF process, details explained in Note S9 (Supporting Information). To avoid the short‐circuit, an electrically insulative Kapton tape was used to cover the patterned doctor blade (Figure 1d). The control of the movement of the doctor blade was governed by a 5‐axis home‐built gantry system (PI mikromove). The μ‐EF casting was done at different thickness to make different thickness of the samples (Figure S7, Supporting Information). A thickness reduction of the teeth and increase in the base height was observed and so a relatively thicker casting thickness was used to make the μ‐EF electrodes. Therefore, a casting thickness of 1100 µm in total with a teeth thickness of 1000 µm and base thickness of 100 µm, was maintained during the μ‐EF casting process. Details of the thickness relationship can be found in Note S6 and Figure S7 (Supporting Information) shows the optimized thickness of the casting.

### Analysis of the Paste Properties and Porosity

The porosity of the electrodes controls the electrochemical performance of the battery and conventionally the porosity was controlled by the calendaring process. However, the calendaring process destroyed the structure of the μ and μ‐EF electrodes. This study avoids the calendaring process for this reason. However, the control of the paste concentration and viscosity was optimized for making this type of thick‐structured electrodes, as demonstrated in the previous report.^[^ ^5^, ^8^ ^]^ As the structure of the electrodes remained similar, this study considered the optimized 40% solid loading for the paste.^[^ ^8^, ^9^ ^]^ This solid loading of the paste achieved sufficient porosity in the electrodes and helped to obtain the necessary structural integrity and sustainability. In this paper, the effect of electric field and drying method on porosity was reported.

### Porosity and Thickness Measurement

The pastes were prepared with solvent (NMP) and the solvents were evaporated during the drying process. To measure the porosity, first the mass and the volume of the electrodes were measured by using weight balance and digital calipers. Digital calipers were also used to measure the thickness of the assembly. The density of the materials used in the paste making are known from the materials specification sheets provided by the vendors. The volume was calculated by using the measured mass, density and composition of the paste and the actual volume was measured using the calipers. The porosity was then calculated from the measured volume and the calculated volume. Another approach of measuring the electrode porosity was using the SEM images of the electrodes at different magnification and then used ImageJ software to process the images to evaluate the porosity of the electrodes.

### Drying of Hyper‐Thick Electrodes

Due to excessive thickness, the drying process needed to be updated and two different methods were optimized. The first method used flash sintering (Xenon lamp, Sinteron 2000), ultra‐violet (UV) light drying, and conventional hot plate drying where all of these had different pros and cons on their own. The overheating using the hot plate (>80 °C) resulted in curving of the thick electrode and flash sintering (30 pulses, voltage of 3 kV, pulse duration 0.2 ms) ended in cracks and breaking of the electrode. One possible way to overcome this was using environmental evaporation of the solvents however this prolonged the drying time (> 3 days) significantly. Therefore, an urge to optimize the process resulted in a heating profile showed in Figure 1h. This optimization was different for anode slurry and cathode slurry due to the difference in active materials. More detailed analysis had been done before optimizing the flash sintering based drying process, showed in Note S7 (Supporting Information). In this method, the drying property also depended on the viscosity of the slurry and compositional ratio of the active materials, binder and conducting agent. Therefore, it will be required to perform another optimization of the drying process if the paste properties will be changed. To introduce a convenient and straightforward way, a laser drying process was used which was a combination of environmental drying, hot plate drying and laser drying method. A fiber laser of wavelength of 976 nm was used to conduct this experiment. A range of laser power (0 W to 40 W) and scan speed (5 to 40 mm s^−1^) were applied to find the optimal parameters. The fastest route for drying was found at 30 min of laser drying with 30 W power and 20 mm s^−1^ of scan speed. Detailed analysis of the laser drying process was also investigated and discussed in Note S8 (Supporting Information).

### Cell Assembly and Test

Afterward, the electrodes were cut to fit within a CR 2032 type coin cell casing (Wellcos Corp.), where the diameters of the cathode and anode electrodes were 14 and 16 mm, respectively. The electrodes were punched using a puncher to cut in circular shape. The cathodes were prepared with a loading of 64.97 mg cm^−2^ and anodes were prepared with a loading of 60.48 mg cm^−2^. The coin cell assembly was done inside a moisture and oxygen‐controlled argon‐filled glove box (MBRAUN). A 25 µm thick commercial PP/PE/PP (poly‐propylene/poly‐ethylene/poly‐propylene) membrane (Celgard) was used as the separator and 0.5 ml of electrolyte, 1 M LiPF_6_ (Lithium hexafluorophosphate) in EC: DMC (ethylene carbonate and dimethyl carbonate) 1:1 (Sigma‐Aldrich) was added in one cell. The cell components (stainless steel (SS) spacer, SS spring, SS top casing, and SS bottom casing), electrodes (anode and cathode with attached current collectors), and separator were sequentially placed inside the coin cell. To ensure the alignment of the structures, the thickness of the anode, separator, and cathode was individually measured before careful assembly and the thickness of the final assembly was checked. If the alignment was not correct, then the measured thickness of the assembly would be higher than the combined thickness of anode, separator, and cathode. By checking the total thickness of anode, separator and cathode when assembled together, the alignment issue was solved. The electrochemical behavior of the assembled batteries was measured by using a battery testing station (NEWARE) at a voltage range between 2.8 to 4.2 V according to the recommended values from the raw materials vendor. The specific capacity and areal capacity were measured at different current densities to observe the performance under different current loads. Potentiostatic battery impedance was also measured at a frequency range of 10 mHz to 100 kHz at a sinusoidal amplitude of 10 mV via electrochemical impedance spectroscopy (EIS) using a potentiostat (Biologic). All the fabricated structures were examined via two different scanning electron microscopies (SEM), one was Helios Hydra (thermoFisher) and Table‐Top SEM (Hitachi TM 1000). The digital photographs were taken by a digital camera. GITT test was done using NEWARE battery tester at a voltage range of 4.2 V for charging and 2.8 V for discharging where all the charging steps were for 10 min and a resting time for each step was 10 min. For GITT measurement, all the cells were from Class A to compare the diffusion coefficient. The charging and discharging current were 0.1C for GITT. The diffusion coefficient was calculated according to the Equation S1 (Supporting Information).^[^ ^49^, ^50^ ^]^ The mechanical properties were evaluated by using a hydraulic press (Strongway). First, the anode, cathode, and separators were assembled together, and then different loads (0 to 2 kN) were applied from top and bottom of the assembly (Figure 6g). The thickness of the assembly was measured by a digital caliper. The initial thickness (at load = 0 kN) was measured before applying any load. X‐ray photoelectron spectroscopy (Thermo Scientific Nexsa) was performed on the structured samples to understand the drying mechanism influence on the chemical composition of the electrode surfaces using a monochromated Al Kα X‐ray source. The spectra of interest were the C1s, F1s, and O1s scans for both the NMC622 and MCMB electrodes. All processing and curve fitting of the data was performed using CasaXPS software.

## Conflict of Interest

The authors declare no conflict of interest.

## Supporting information



Supporting Information

## Data Availability

The data that support the findings of this study are available in the supplementary material of this article.
