# Supplementary material for: Hyper‐Thick Electrodes for Lithium‐Ion Batteries Enabled by Micro‐Electric‐Field Process
Source: Adv Sci (Weinh). 2024 Dec 25;12(7):2413444. doi: 10.1002/advs.202413444 (PMC11831531; doi:10.1002/advs.202413444)
Supplement: Supplementary file 1 — Supporting Information [file ADVS-12-2413444-s001.pdf]

ADVANCED SCIENCE
Open Access

Supporting Information

for *Adv. Sci.*, DOI 10.1002/advs.202413444

Hyper-Thick Electrodes for Lithium-Ion Batteries Enabled by Micro-Electric-Field Process

*Tazdik Patwary Plateau, Gracie Boyer and Jonghyun Park**

Supplemental Information

Hyper-Thick Electrodes for Lithium-Ion Batteries Enabled by Micro-Electric-Field Process

Tazdik Patwary Plateau[1], *Gracie Boyer*[1], *Jonghyun Park*[1,2]*

[1]Department of Mechanical and Aerospace Engineering

[2]Department of Electrical and Computer Engineering

Missouri University of Science and Technology

Rolla, MO 65409, United States

* parkjonghy@mst.edu

Keywords: hyper-thick electrodes, micro-macro diffusion path design, active materials particle arrangement, electric-field casting, electrode structure engineering

Supplemental note 1 – Effect of electric field on the particles in paste solution

To observe the effect of the electric field on the particles, a test was conducted where two metal pieces were used. Previously in literature, a study showed the active material particles movement while applying high voltage electric field [9]. In that study, dry $LiMn_2O_4$ (LMO) particles were placed on a paper and an electric field was conducted on the particles. The results showed the particles kept aligned due to the electric field. However, it was not observed how the particles move in a slurry containing active material, binder, conductive agent, and solvent when an electric field was applied. In this work, we conducted an experiment to observe the particle movement in the slurry using a high voltage electric field of 3 kV. As the active materials are not dielectric materials, a high electric field had to maintain for inducing polarization in the anode and cathode particles. The EF applied during casting caused the active material particles to align, resulting in improved particle arrangement.[9] Moreover, in the slurry system, the electric field can influence these particles through alternative mechanisms, such as charge redistribution or particle-medium electrostatic interactions, facilitating their alignment. These combined effects, including dielectric polarization and electrostatic interactions, likely explain the observed structural changes [8, 23-24]. Therefore, the particle alignment is driven by a combination of dielectric polarization, charge interactions, and electrostatic forces, rather than purely classical dielectric behavior. According to **Figure S1a**, two metal pieces were connected to a high voltage of 3kV. One of the metal pieces was covered by the Kapton tape to avoid short circuit while in contact with the slurry material. To get a clear observation, a dilute solution of the slurry material containing 85.5% NMC-622 as active materials, 6.5% carbon black, 8% PVDF binder in the NMP solvent was used. The metal pieces were placed with a gap of 500 μm between them. The slurry was then dropped in between the gap using a dropper. **Figure S1b-j** show the movement of the particles in the slurry. The black colored particles were the solid particles. After using 3 kV for 0.8 second, the particles tended to form linear structure. This may attribute to the presence of extremely high electric field in between the doctor blade and the substrate. This experimental observation indicated the ability of arranging the particles in the electrode.

Supplemental note 2 – Delamination issue

One method to resolve the delamination issue is to increase the binder amount. However, increasing binder amount (PVDF, polyvinylidene fluoride) reduced the electrical conductivity (**Figure S8**). Adding conducting agent carbon black (CB) in the paste slurry enhanced the electrical conductivity. But adding both PVDF and CB reduce the percentage of the active material in the electrode which is not desired. Therefore, we tried another route to improve the PVDF by pre-drying of the powders (95°C for 10hours). In addition, the current collector foils were also treated to enhance the adhesion properties. Due to very thin film of foils (aluminum foil thickness 12.5 μm and copper foil thickness 10 μm), the mechanical etching could not be achieved (**Figure S9**). So, acid based etching process has been optimized by using different acids with different concentrations of them (showed in **Table. S1**). First, three common acids for acid etching was chosen – HCl, H2SO4 and H3PO4. We determined the contact by measuring conductivity showed in **Figure S10**. By evaluating the results showed in **Figure S11**, it is clear that the 5% HCl solution showed the best conductivity which ensures the contact between the electrode and the current collector. A comparative image between the etching effect has been showed in **Figure S12**.

Supplemental note 3 – X-ray photoelectron spectroscopy

X-ray photoelectron spectroscopy (Thermo Scientific Nexsa) was performed on the structured samples to understand the drying mechanism influence on the chemical composition of the electrode surfaces using a monochromated Al Kα X-ray source. The spectra of interest were the C1s, F1s, and O1s scans for both the NMC622 and MCMB electrodes. All processing and curve fitting of the data was performed using CasaXPS software.

Figure S13 shows the C1s, O1s, and F1s spectra for the laser and flash sintered cathode samples. The sp^2 peak was fitted with an asymmetric Lorentzian line shape LA (2.0,2.5,5) shape due to the graphitic nature of the C=C bonds present within the electrodes[28]. A symmetric line shape of SGL (20) was used for the remaining signals in the C1s spectra, F1s spectra, and O1s spectra [29]. Reference binding energy and FWHM for components of the C1s spectra for carbon contamination, graphite, and carbon black were obtained from Biesinger[28]. Reference binding energy of the PVDF (-CH_2-CF_2-) C1s and F1s signals were obtained from Chastain, et al. and Chiang, et al.[30,31]. Before curve fitting, a Shirley background was applied to all spectra. Calibration for all sample spectra was performed using the 284.5 eV C=C peak. **Tables S4, S5**) contains the BE reference values, the sample BE values, and FWHM used during the curve fitting process. For the laser dried and flash sintered samples, the C1s signals consist of sp2 hybridization (284.5 eV), C-C, C-H (248.8-285 eV), followed by the PVDF signals: CH_2 (286-286.13 eV) and CF_2 (290.57-290.69 eV), oxygen containing contaminants (C-OH, C-O-C at 286.54-286.69 eV, C=O at 287.93-288 eV, and O-C=O at 289.06-289.25 eV), and a final pi-pi* shake-up satellite peak 292.52-292.67 eV). A variation in the amount of PVDF within the signal can be seen in **Figure S13c**, where the laser dried cathode shows CH_2/CF_2 primary peaks while the primary peak in the flash sintered cathode is the C=C peak. The F1s spectra for both cathode samples showed a single peak, resulting from the CF_2 signal from PVDF (687.7-687.8 eV). The O1s spectra for the cathodes show three peaks resulting from the hydrocarbon contaminants (C=O at 531.56-531.62 eV, C-O at 533.44-533.62 eV) and the lattice oxygen within the $LiNi_6Mn_2Co_2O$ samples (529.56-529.65 eV). Though curve fitting of the cathode C1s spectra shows a difference in the PVDF and C=C amounts between the laser dried and flash sintered samples, and a variation in the ratio of C-O to C=O, there are not any additional peaks introduced in either the laser or flash sintered samples, concluding that the drying techniques do not change the chemical composition of the cathodes. Curve fitting of the

anode spectra for both laser dried and flash sintered electrodes shows contributions from graphite (284.5 eV), C-C, C-H (248.8 eV), PVDF (CH_2 at 286.04-286.12 eV, CF_2 at 290.62-290.7 eV), oxygen containing species (C-OH, C-O-C at 286.56-286.61 eV, C=O at 287.92-288 eV, and O-C=O at 289.17-289.3 eV), and a final pi-pi* shake-up satellite peak (291.88-291.95 eV). The overlaying of the laser dried and flash sintered MCMB samples in **Figure S14** show similar C1s, F1, and O1s spectra with no additional peaks resulting from either drying technique, confirming the drying process does not influence the chemical composition for the graphite-based electrodes.

Supplemental note 4 – Impedance analysis

The impedance analysis was evaluated by using the EIS (electrochemical spectroscopy) testing. Two types of cells were used for this test – μ cell and μ-EF cell. **Figure 5a** showed the circuit diagram used in this experiment. The naming of each component of the circuit diagram is as follow,

Re: resistance of bulk electrolyte

Rsl: surface layer resistance

Rct: charge transfer resistance

Csl: surface layer capacitance

Cdl: non-far-adaic-branch consisting of the double layer capacitance

Zw: Warburg impedance

ZFSW: finite space Warburg-type element

The occurrence of double layer capacitance is a result of the presence of different charge carriers in materials, with electrons in the metal and ions in the fluid. The charge carried by the metal attracts ions from the fluid to the metal-fluid interface, giving rise to the double layer capacitance (C_{dl}). Through repeated cycling, a surface layer forms and expands at the electrode-electrolyte interface, leading to the capacitance and resistance of this surface layer, referred to as C_{sl} and R_{sl}, respectively. In impedance spectroscopy, a finite space Warburg-type element (Z_{FSW}) is employed to describe diffusion in a medium where the interface restricts the flow of species. This element is represented by the hyperbolic cotangent function. [27] Typically, two semicircles can be observed in a Nyquist plot: the low-frequency semicircle corresponds to the charge transfer resistance (R_{ct}) [38], while the high-frequency semicircle relates to the kinetics of the electrochemical reaction, which can be influenced by factors like surface coating, phase transitions, bandgap structure, and particle size. [38] The long tail of the Nyquist plot, referred to as the Warburg tail, is associated with the diffusion of ions. In this specific case, the first semicircle (R_{ct}, shown in **Figure 5b-c**) is visible at low frequency, but the second semicircle at high frequency is not observed. This absence may be attributed to the dominant effect of high ion diffusion, as indicated by the steepness of the Warburg

tail slope. Diffusion becomes challenging as it approaches a 45° angle with the real impedance (Z') axis.

Supplemental note 5 – GITT analysis

The GITT test was conducted at a very low C-rate of 0.01C to observe the charging and discharging. Each step was for 10 minutes interval. As showed in **Figure 5f**, the voltage increased when an extremely low current was applied and dropped during the resting of the cell. The full voltage profile can be seen is Figure 5e and a tiny portion of the voltage profile was illustrated in **Figure 5f**. The change in voltage was calculated for the transient state ΔE_t and steady-state voltage difference ΔE_s from the voltage profile. These values were used to calculate the diffusion coefficient using **Equation S1**,

$$D = \frac{4}{\pi\tau}\left(\frac{n_m V_m}{S}\right)^2\left(\frac{\Delta E_s}{\Delta E_t}\right) \qquad \text{(S1)}$$

The above equation denotes the parameter such as

D: diffusion coefficient

τ: duration of the current pulse (10 minutes = 600 s)

n_m: number of moles (mol)

V_m: molar volume of the electrode (cm^3 / mol)

S: electrode/electrolyte contact area (cm^2)

ΔEs: steady-state voltage change due to current pulse

ΔEt: voltage change during the constant current pulse

The diffusivity was plotted at different voltages in **Figure 5d** where it can be observed that the diffusivity values were higher for the µ-EF battery than the µ battery. This high diffusivity may be attributed to the benefits of well arrangement of the active material particles in the electrodes, enabling facile ion diffusion.

Supplemental note 6 – Casting thickness and electrode thickness

During the drying process, the evaporation of the solvent was not uniform from all the regions of the electrodes. Because of the high thickness of the electrode, the inner parts of the electrodes were more unfavorable to evaporate solvents than the surface parts of the electrodes. Therefore, collapsing of the materials happened during the drying process which resulted in two distinct phenomena. One is the curvature on the top of the teeth of the pattern (showing in **Figure S7a** to **Figure S7b**). Second is the reduction in the thickness of the teeth and enhancement of the base thickness as showed in the schematic diagram of the cross-section in **Figure S10c-e**. From the experimental observation related to the thickness of the base and teeth is showed in **Figure S10f** (thickness after casting) and **Figure S10g** (thickness after drying). In this analysis, all the drying processes followed the flash sintering based drying process. In summary, to make a hyper-thick electrode of 700 μm in total thickness (550 μm teeth and 150 μm base), the casting was required to have 1100 μm with a teeth thickness of 1000 μm and base of 100 μm.

Supplementary note 7 – Optimization of flash sintering based drying method

The high thickness of the electrodes led to complications during the drying process. Specifically, a significant thermal gradient formed inside the electrode during the traditional vacuum oven based drying method. The conventional approach of drying was conducted at 120 ^{o}C overnight in vacuum. This leads to the exposure of the heat flux around the electrodes, but the core of the electrode remained dry. Another option was environmental drying from ambient evaporation of the solvent. However, this process took more than 3 days to fully dry the electrode. Therefore, it was required to find an optimized drying process for the thick electrode. This study investigated two different heating mechanisms – flash sintering based method and laser drying method. Both of these methods were very fast and high temperature drying techniques. Thus, the processes were combined with different slower methods like hot plate drying, UV drying and ambient drying process. First the flash sintering based drying process was optimized. **Figure S5** showed the impact of the flash sintering pulses on the thick electrodes. Here the casting thickness was 1000 μm. Increasing the pulses of the flash sintering process can dry the electrode, however, it generated large cracks leading to possible breaking of the electrodes. **Table S2** showed the effect of different voltage used in the flash sintering process. Observing the experimental results of the maximum achievable thickness and resistivity, it was concluded that the 3kV voltage showed better results. However, this could not solve the problem of breaking and cracking of the electrodes. In addition to the flash sintering, a UV drying process was introduced which simultaneously dried the electrode with a UV light while the sample was heated on a hot plate.   The high temperature of the hot plate resulted in curving of the electrodes and delamination from the current collector. The best drying method was environmental drying without any heat source however it took more than 3 days to dry the electrode completely. Therefore, an optimal heat profile was required. After several trial-and-error, it was possible to make the electrode with any delamination, fracture, cracking or breaking of the electrode. The achieved heating profile was showed in **Figure 1j**.

Supplementary note 8 – Optimization of laser parameters

6 different laser parameters were optimized: (1) laser power, (2) laser scan speed, (3) distance between the laser source and the electrode, (4) frequency, (5) pulse rate and (6) environment. Additionally, the environmental influence was tested with both air and nitrogen, however there was no significant change in the conductivity and electrode structure. For convenience, air atmosphere was chosen. The longer pulse rate ended in burning and cracking on the electrode and very short pulses could not generate the power to evaporate the solvent. An optimal pulse rate of 20 μs was applied to the electrodes for drying. Furthermore, the frequency of the laser was also evaluated. It was found that the higher frequency tended to penetrate the electrode, resulting in the formation of holes. Therefore, the frequency <100 Hz showed suitable results in terms of electrode morphology. The distance between the laser source and electrode was set at the focal length of laser to achieve the maximum energy to dry with the laser system. The major influence on the electrode characteristics was from the laser power and laser scan speed. Therefore, a thorough investigation had been conducted to observe the relationship between the laser power and holding time. It was found that the high laser power and longer holding time resulted in burning of the electrode. Different observations were illustrated in **Figure 1m** in the main text. Similar investigation was done to evaluate the impact of the scan speed over the exposure time of the laser on the electrode slurry. It was found that the longer exposure and slower scan speed could burn the electrodes instead of drying them. **Figure 1n** showed the detailed results observed from the experiments.

Supplemental note 9 – High electric field in μ-EF casting

Though higher voltages could have resulted in the further improvement of the particle arrangement within the electrodes, the small separation (150 μm) between the doctor blade assembly and the grounding plate (Kapton tape: 50 μm, minimum electrode slurry thickness: 100 μm) restricted the casting voltage to 3 kV. Voltages greater than 3kV resulted in spark formation, leading to undesirable burning of the electrode surface.

Table S1. Different acid samples for acid etching of current collector

Sample no.	Acid	Concentration percentage
1	H_2SO_4	10
2	H_3PO_4	10
3	HCl	10
4	HCl	5
5	HCl	15
6	HCl	20

Table S2. Flash sintering voltage and achieved maximum thickness of the electrodes and their resistivity.

Voltage (kV)	Maximum Achievable Thickness (micron)	Resistivity (kohm/cm)
1.75	910	5.5
2	960	2.61
2.25	980	0.615
2.5	1020	0.628
2.75	1060	0.308
3	1200	0.245

Table S3. Results of porosity in the electrodes fabricated by Laser drying process and Flash sintering based drying process. The calculation has been done by using ImageJ software utilizing the SEM images of the electrodes.

	**Flash sintering**	**Laser**
**Average porosity**	0.424	0.55
**Max porosity**	0.443	0.597
**Min porosity**	0.379	0.486

Table S4. Curve Fitting Parameters - C1s [28-31]

		**Laser Dried Cathode**		**Flash Sintered Cathode**		**Laser Dried Anode**		**Flash Sintered Anode**	
**C1s Signals**	**Reference Binding Energy (eV)**	**Binding energy (eV)**	**FWHM**	**Binding energy (eV)**	**FWHM**	**Binding energy (eV)**	**FWHM**	**Binding energy (eV)**	**FWHM**
C=C (sp2)	284.5	284.5	0.73	284.5	0.68	284.5	0.69	284.5	0.64
C-H,C-C	284.8-285	285	1.45	284.8	1.5	284.8	1.5	284.8	1.48
C-OH, C-O-C	286.3-286.7	286.54	1.45	286.69	1.5	286.56	1.5	286.61	1.48
C=O	287.8-288	287.93	1.45	288	1.5	287.92	1.5	288	1.48
O-C=O	288.8-289.3	289.06	1.45	289.3	1.5	289.17	1.5	289.3	1.48
pi to pi sat	290.91	292.67	2.6	292.19	2.6	291.95	2.6	291.88	2.6
CF2	290.8	290.57	1.23	290.68	1.25	290.62	1.19	290.7	1.18
CH2	286.3	286	1.23	286.12	1.25	286.04	1.19	286.12	1.18

Table S5. Curve Fitting Parameters - F1s and O1s [28-31]

		**Laser Dried Cathode**		**Flash Sintered Cathode**		**Laser Dried Anode**		**Flash Sintered Anode**	
**O1s Signals**	**Reference Binding Energy (eV)**	**Binding energy (eV)**	**FWHM**	**Binding energy (eV)**	**FWHM**	**Binding energy (eV)**	**FWHM**	**Binding energy (eV)**	**FWHM**
Lattice O	529.6-529.7	529.56	1.96	529.65	1.73	N/A	N/A	N/A	N/A
C=O	~531.7	531.62	1.96	531.56	1.73	531.89	2.32	531.69	2.4
C-O	~533.4	533.62	1.96	533.44	1.73	533.15	2.32	533.12	2.4
**F1s Signal**									
CF2	687.8	687.7	1.89	687.81	1.9	687.73	1.87	687.77	1.86

Table S6. Electrochemical impedance spectroscopy parameters used for **Figure 5b**.

**Parameters**	**µ**	**µEF**
**R_e, Ω**	7.29	4.83
**R_{ct} + R_{sl}, Ω**	23.96	10.52
**C_{dl}, F**	3.34×10^{-7}	3.795×10^{-6}
**C_{sl}, F**	9.133×10^{-9}	8.98×10^{-9}
**Z_w, Ω $s^{-1/2}$**	119.76	80
**Z_{FSw}, Ω $s^{-1/2}$**	163.93	43.47

Table S7 Electrochemical impedance spectroscopy parameters used for **Figure 5c**.

<table>
<tr><th>Parameters</th><th>1st</th><th>50th</th><th>200th</th></tr>
<tr><td>R_e, Ω</td><td>7.29</td><td>7.26</td><td>9.14</td></tr>
<tr><td>R_{ct}, Ω</td><td rowspan="2">22.03</td><td>8.5</td><td>10.17</td></tr>
<tr><td>R_{sl}, Ω</td><td>17.67</td><td>22.26</td></tr>
<tr><td>C_{dl}, F</td><td>4.03×10^{-7}</td><td>3.945×10^{-7}</td><td>9.81×10^{-7}</td></tr>
<tr><td>C_{sl}, F</td><td>9.33×10^{-9}</td><td>5.53×10^{-7}</td><td>8.23×10^{-7}</td></tr>
<tr><td>Z_w, Ω $s^{-1/2}$</td><td>86.91</td><td>204.49</td><td>246.913</td></tr>
<tr><td>Z_{FSw}, Ω $s^{-1/2}$</td><td>45.248</td><td>89.25</td><td>102.9</td></tr>
</table>

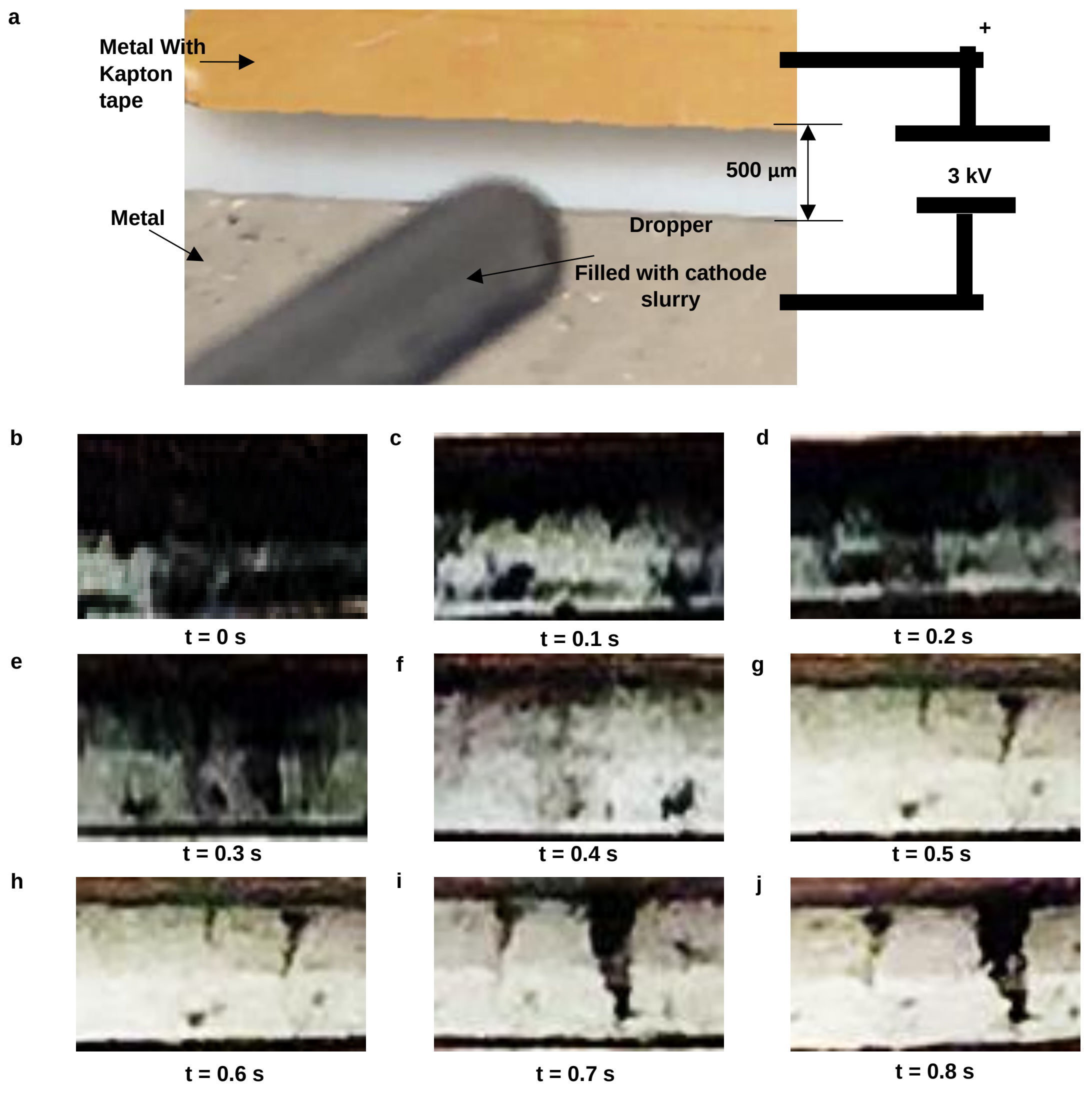

Figure S1. The setup for the experiment to evaluate the effect of the electric field on the particles used in the slurry (a). Images of the particle-arrangement at different time steps from t = 0 s (b) to t = 0.8 s (j).

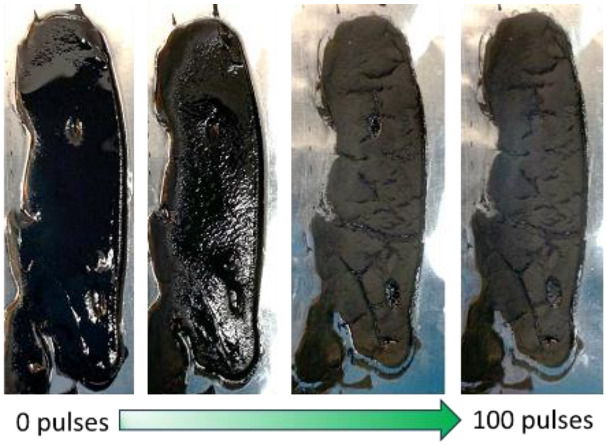

Figure S2. Effect of Flash Sintering on the thick electrode.

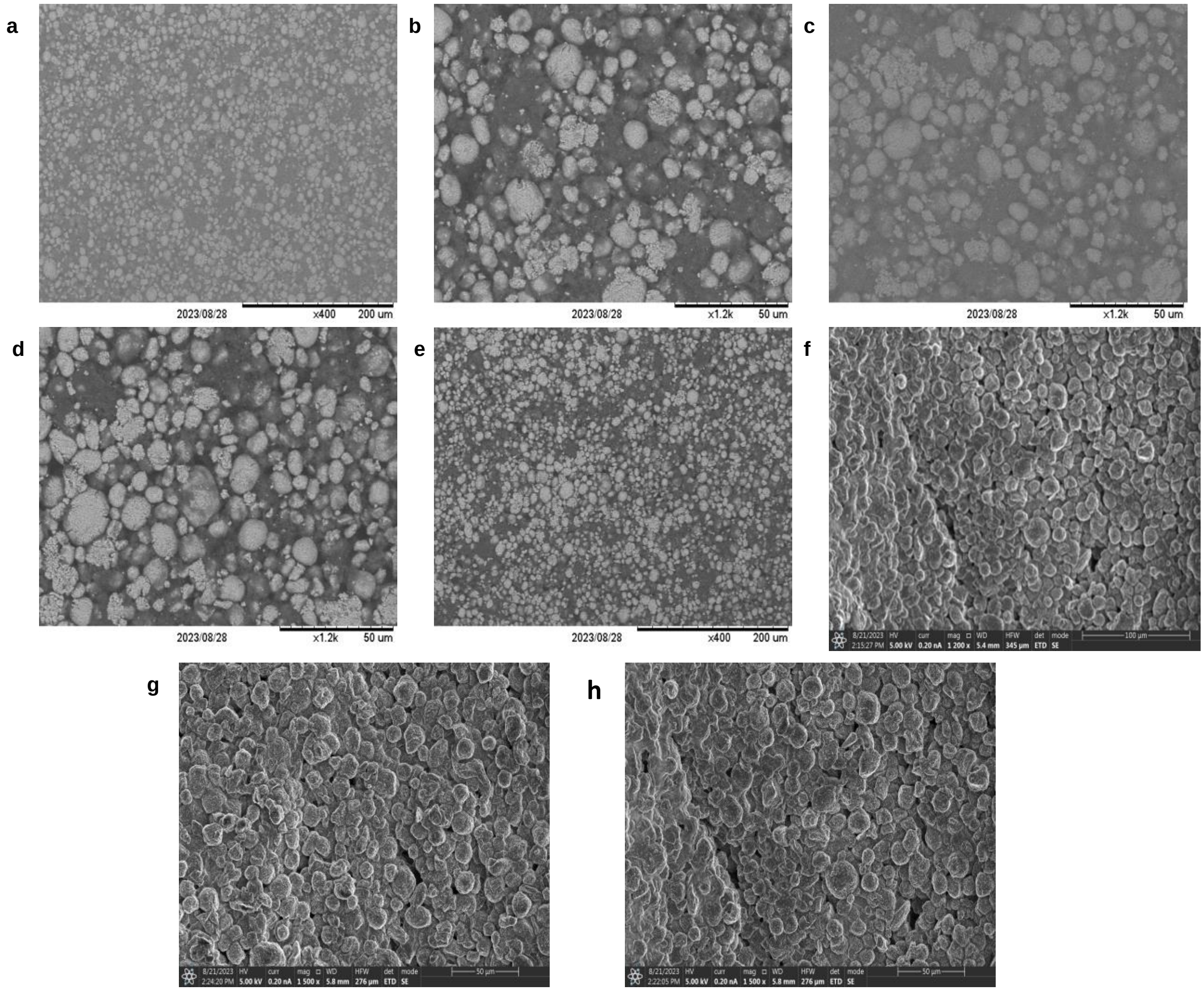

Figure S3. SEM images of different positions in multiple electrodes fabricated using with EF (a-e) and SEM images at the cross-section of the micro-EF electrode (g-h).

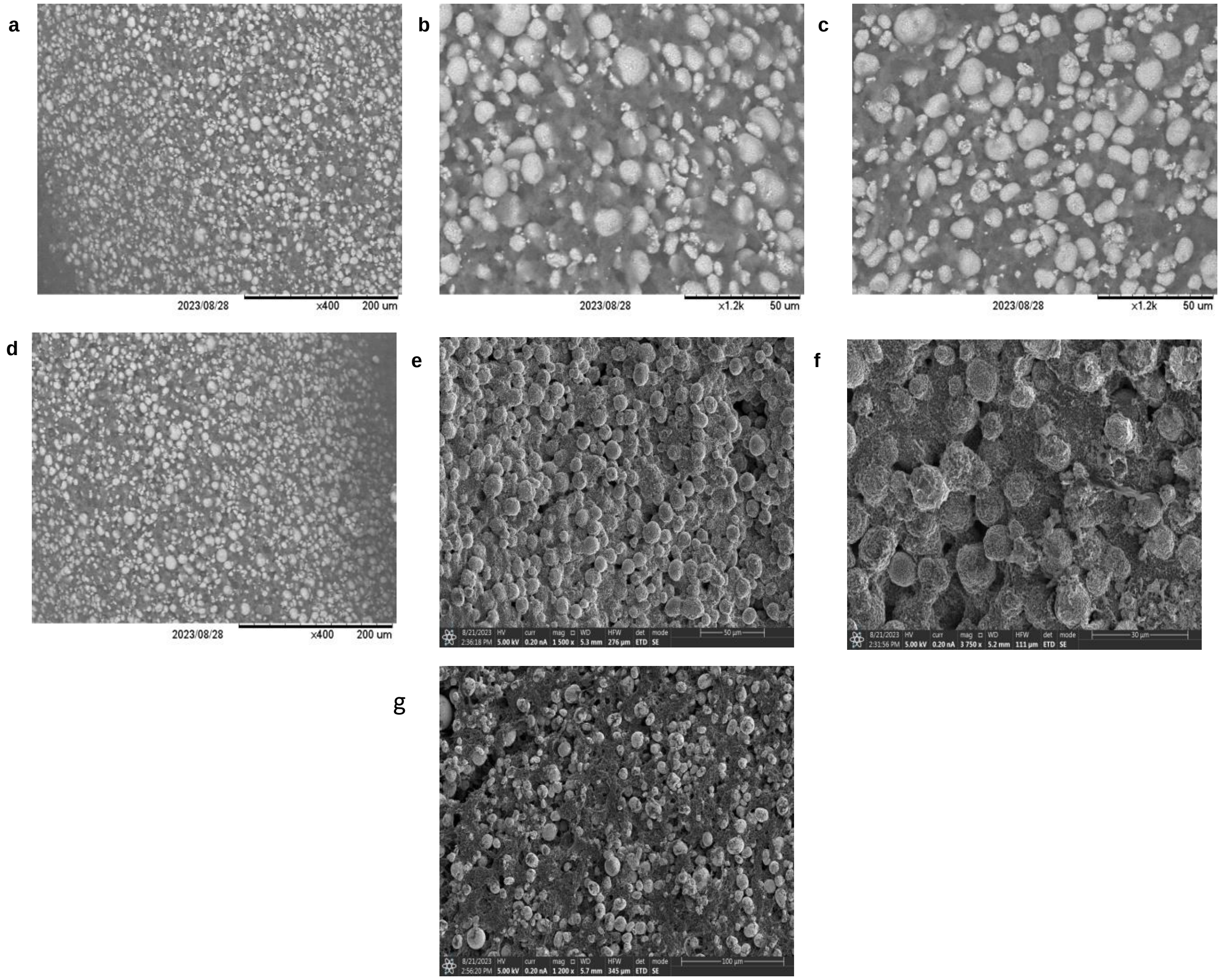

Figure S4. SEM images of different positions in multiple electrodes fabricated without EF (a-d) and SEM images of the cross-section of the electrodes fabricated without EF (e-g).

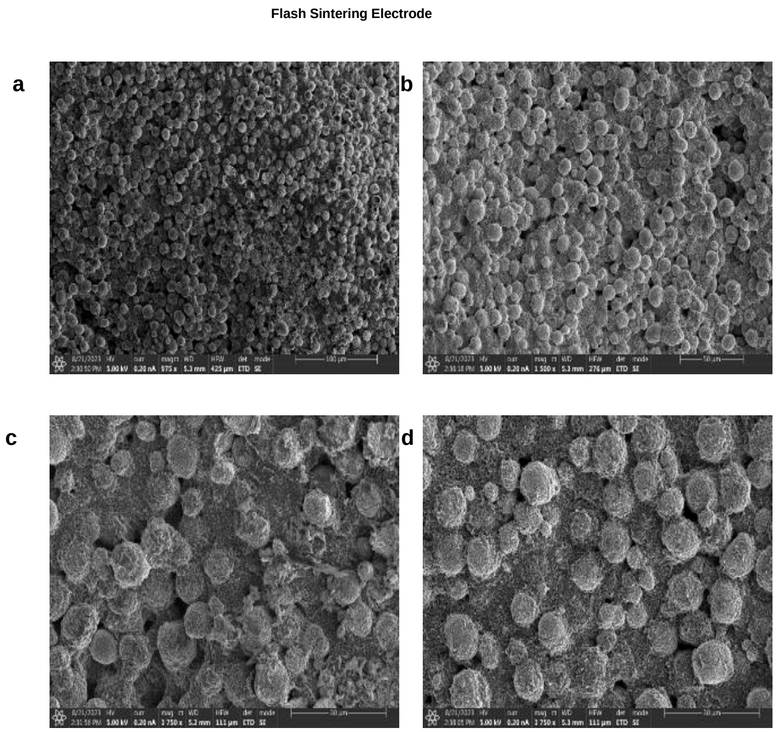

Figure S5. SEM images of electrodes fabricated by using Flash Sintering drying process.

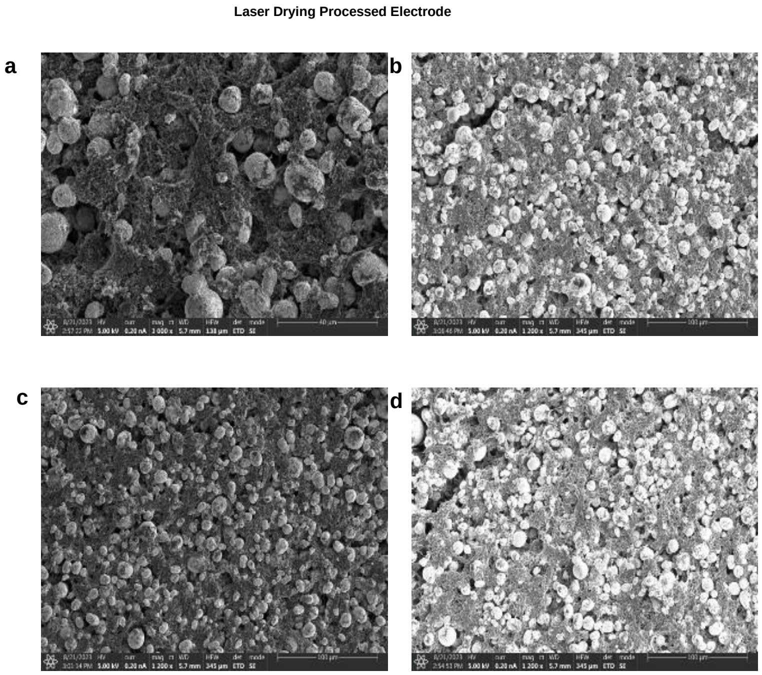

Figure S6. SEM images of electrodes fabricated by using Laser drying process.

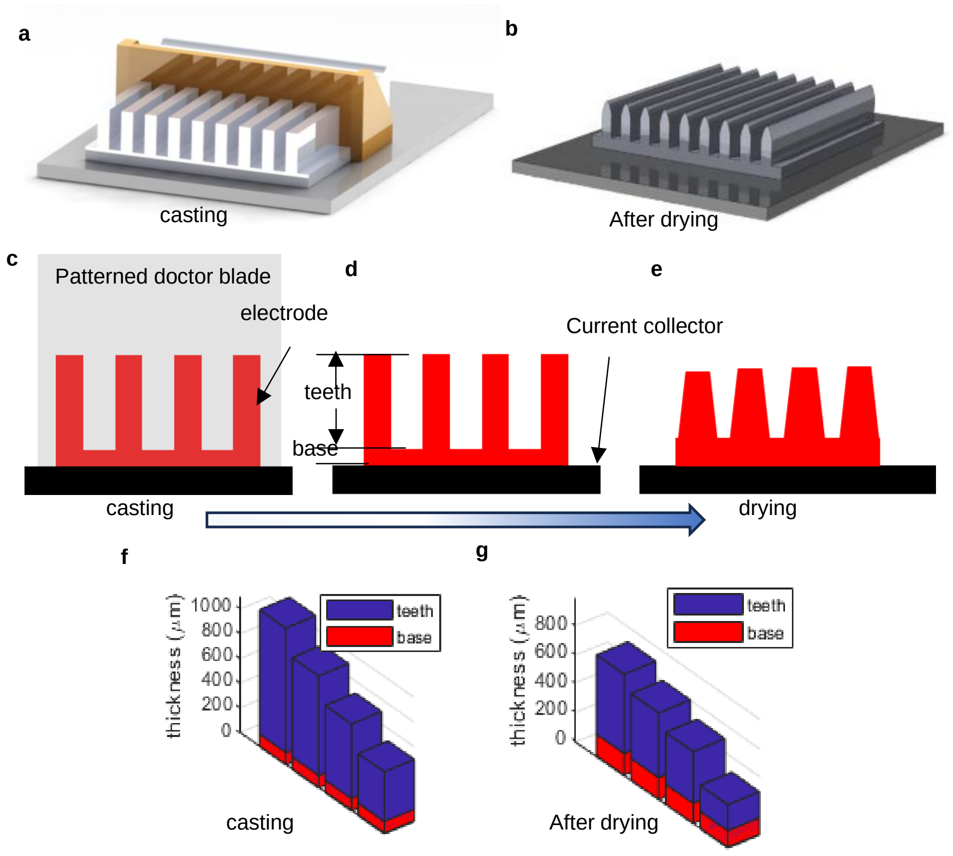

Figure S7. A 3D schematic view of the electrode structure during the casting process (a), a 3D schematic view of the electrodes after drying (b), 2D schematic view showing the cross-sectional view of the electrode structural change (c-e). The casting thickness (f) and thickness after drying (g) where red zone shows the base thickness and blue zone indicates teeth thickness.

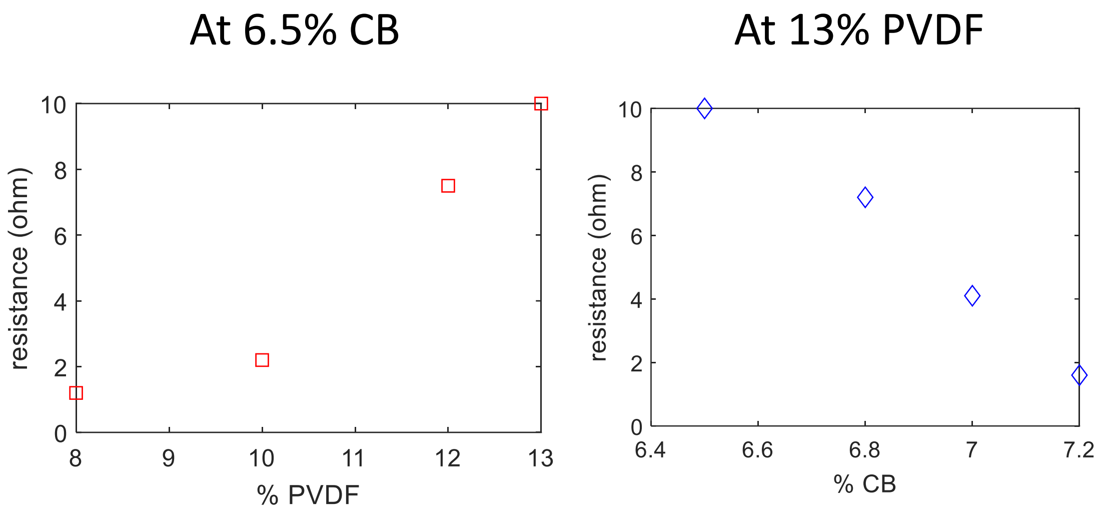

Figure S8. Analysis of the resistance change in the electrode due to the change in carbon black (CB) and binder (PVDF).

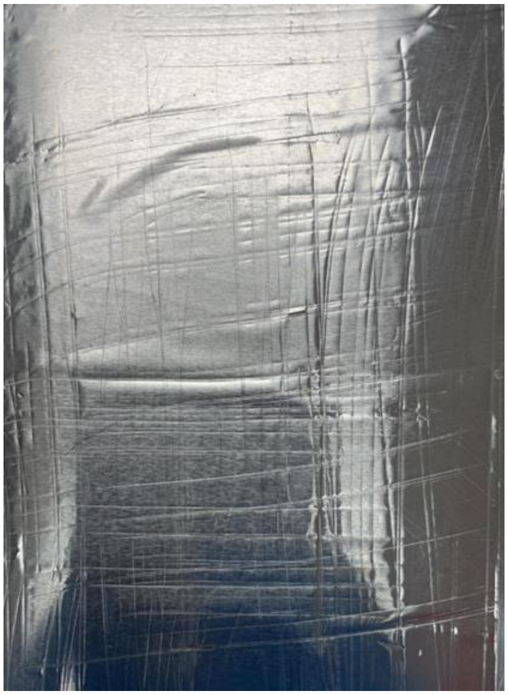

Figure S9. Mechanical etching on the aluminum foil.

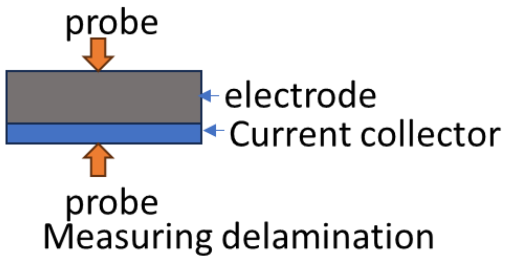

Figure S10. The measurement of delamination of electrode from the current collector by measuring the resistance using a multimeter.

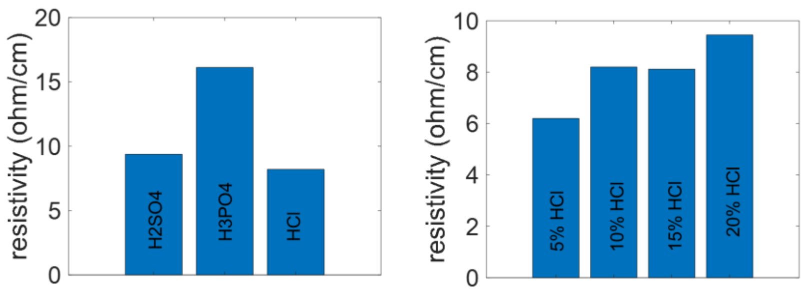

Figure S11. The resistivity measurement results using different acid concentrations for evaluating adhesion of the electrodes.

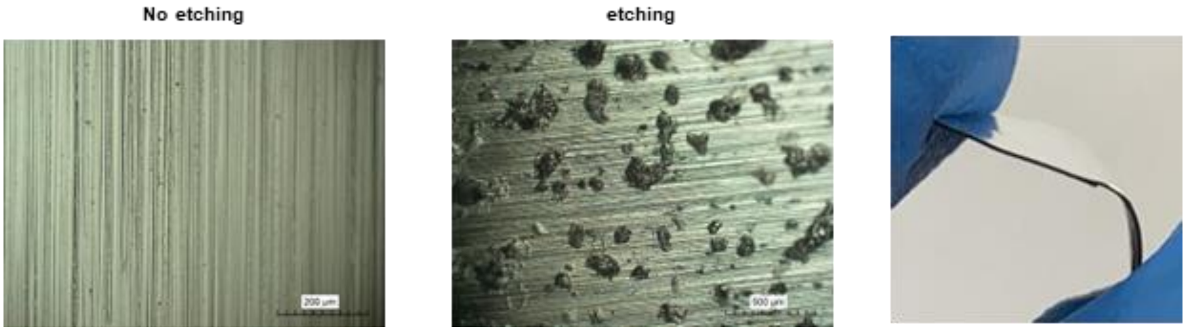

Figure S12. Aluminum foil showing the effect of etching. An image of etched thick electrode with a thickness of 1mm.

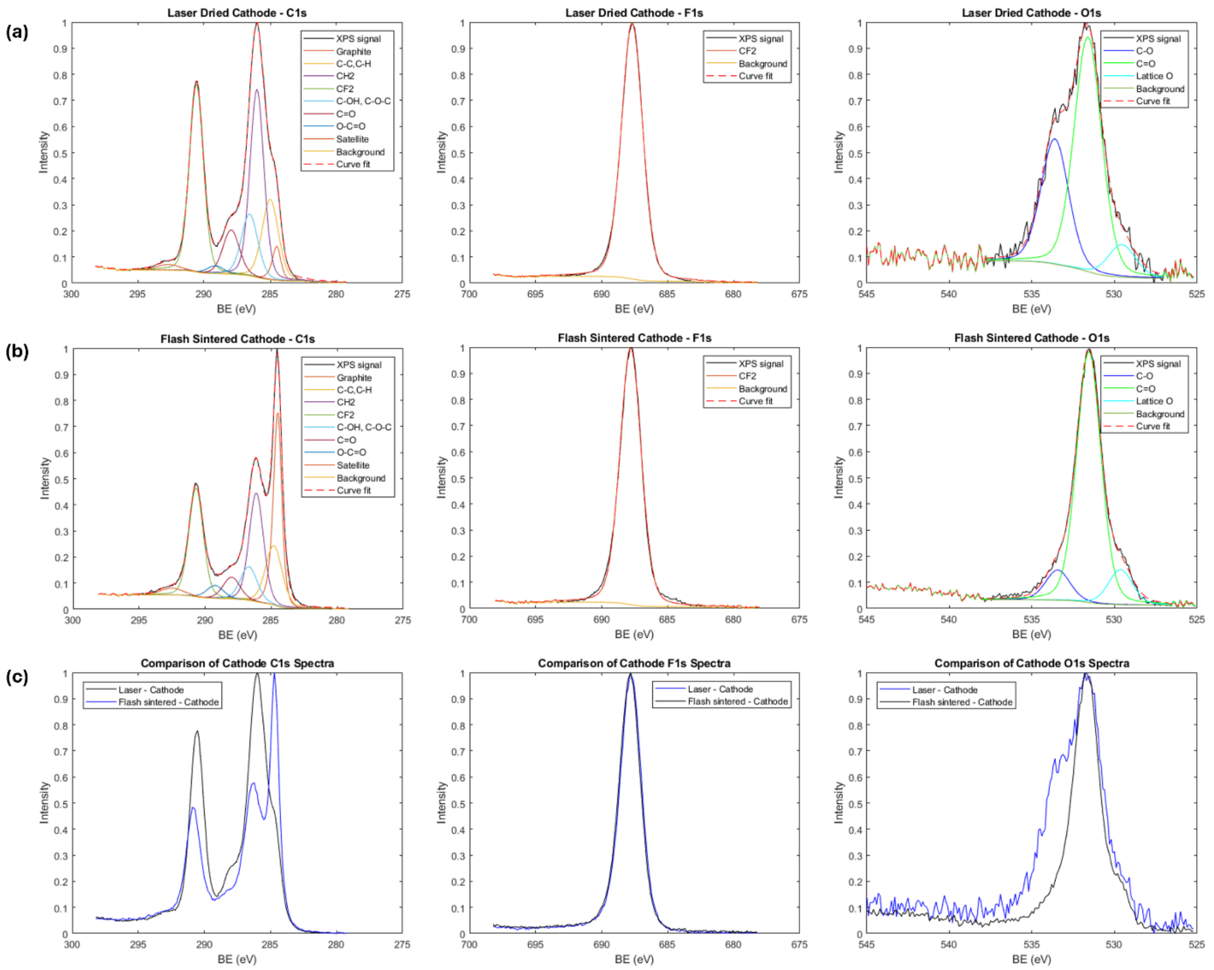

Figure S13. XPS spectra for laser dried and flash sintered cathode samples (a) Curve fitting of laser dried samples (b) Curve fitting of flash sintered samples (c) Comparison of laser dried and flash sintered samples.

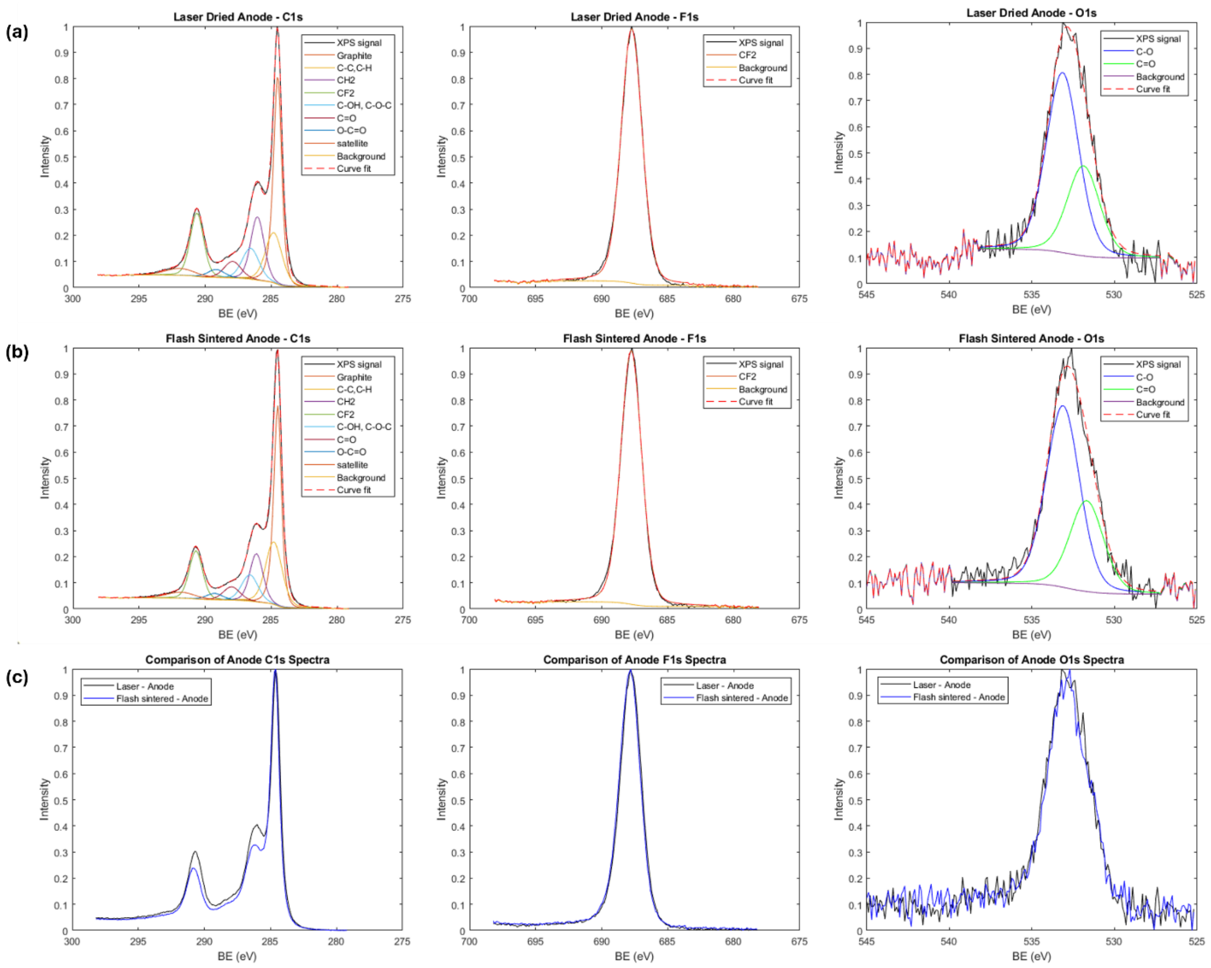

Figure S14. XPS spectra for laser dried and flash sintered anode samples (a) Curve fitting of laser dried samples (b) Curve fitting of flash sintered samples (c) Comparison of laser dried and flash sintered samples.

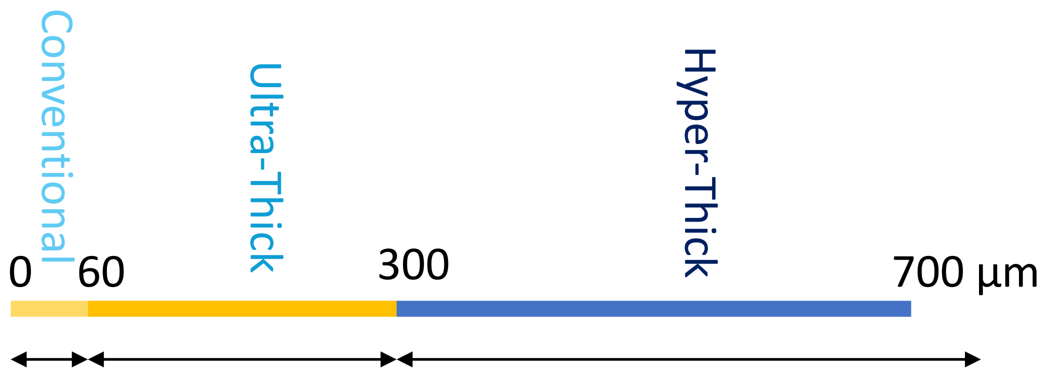

Figure S15. The scale bar defining the electrode names according to thickness unit in µm.

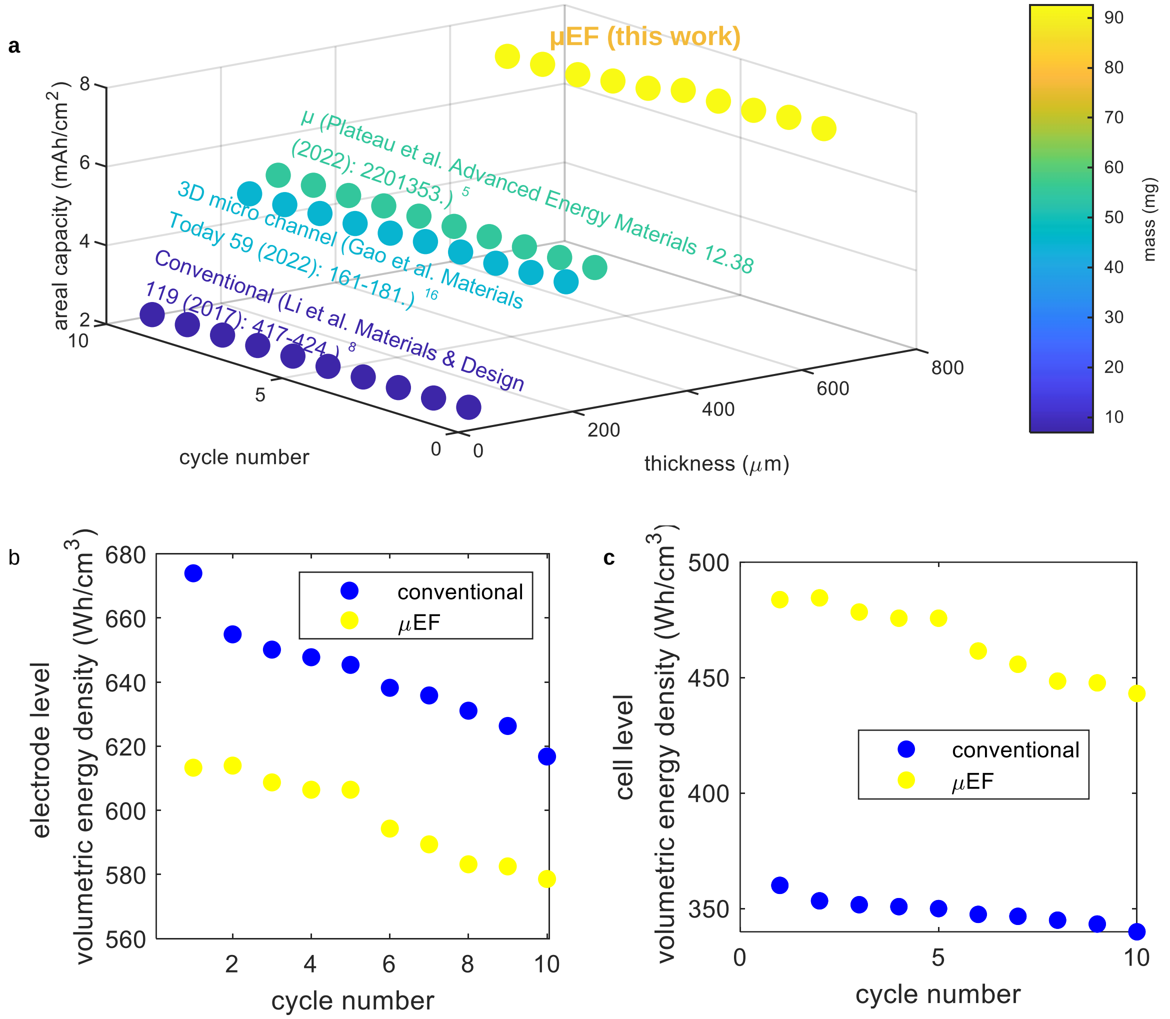

Figure S16. Areal capacity comparison among different 3D structured high energy density thick electrodes where a color bar illustrated the mass of the electrodes according to the thickness for first 10 cycles (a). Here μEF electrode was compared with μ [5], 3D micro-channel [16], and conventional [8] electrodes. The electrode level volumetric energy density comparing graph has been showed in (b) and cell level volumetric energy density comparing graph in between conventional and μEF electrodes depicted in (c).
